# Negative regulation of DNMT3A *de novo* DNA methylation by frequently overexpressed UHRF family proteins as a mechanism for widespread DNA hypomethylation in cancer

**DOI:** 10.1038/celldisc.2016.7

**Published:** 2016-04-12

**Authors:** Yuanhui Jia, Pishun Li, Lan Fang, Haijun Zhu, Liangliang Xu, Hao Cheng, Junying Zhang, Fei Li, Yan Feng, Yan Li, Jialun Li, Ruiping Wang, James X Du, Jiwen Li, Taiping Chen, Hongbin Ji, Jackie Han, Wenqiang Yu, Qihan Wu, Jiemin Wong

**Affiliations:** 1 Shanghai Key Laboratory of Regulatory Biology, the Institute of Biomedical Sciences and School of Life Sciences, East China Normal University, Shanghai, China; 2 Chinese Academy of Sciences Key Laboratory of Computational Biology, Chinese Academy of Sciences-Max Planck Partner Institute for Computational Biology, Shanghai Institutes for Biological Sciences, Chinese Academy of Sciences, Shanghai, China; 3 Key Laboratory of Systems Biology, Institute of Biochemistry and Cell Biology, Shanghai Institutes for Biological Sciences, Chinese Academy of Sciences, Shanghai, China; 4 CAS center for Excellence in Molecular Cell Science, Institute of Biochemistry and Cell Biology, Shanghai Institutes for Biological Sciences, Chinese Academy of Sciences, Shanghai, China; 5 Innovation Center for Cell Signaling Network, Institute of Biochemistry and Cell Biology, Shanghai Institutes for Biological Sciences, Chinese Academy of Sciences, Shanghai, China; 6 School of Life Science and Technology, Shanghai Tech University, Shanghai, China; 7 Laboratory of RNA Epigenetics, Institutes of Biomedical Sciences & Department of Biochemistry and Molecular Biology, Shanghai Medical College, Fudan University, Shanghai, China; 8 Department of Molecular Carcinogenesis, The University of Texas MD Anderson Cancer Center, Smithville, TX, USA; 9 Collaborative Innovation Center for Cancer Medicine, Sun Yat-Sen University Cancer Center, Guangzhou, China

**Keywords:** cancer, DNA, DNMT1, DNMT3A, hypomethylation, UHRF1, UHRF2

## Abstract

Global DNA hypomethylation is a most common epigenetic alteration in cancer, but the mechanism remains elusive. Previous studies demonstrate that UHRF1 but not UHRF2 is required for mediating DNA maintenance methylation by DNMT1. Here we report unexpectedly a conserved function for UHRF1 and UHRF2: inhibiting *de novo* DNA methylation by functioning as E3 ligases promoting DNMT3A degradation. UHRF1/2 are frequently overexpressed in cancers and we present evidence that UHRF1/2 overexpression downregulates DNMT3A proteins and consequently leads to DNA hypomethylation. Abrogating this negative regulation on DNMT3A or overexpression of DNMT3A leads to increased DNA methylation and impaired tumor growth. We propose a working model that UHRF1/2 safeguards the fidelity of DNA methylation and suggests that UHRF1/2 overexpression is likely a causal factor for widespread DNA hypomethylation in cancer via suppressing DNMT3A.

## Introduction

In mammals, DNA methylation at cytosine-C5 in the context of CpG dinucleotides is a key epigenetic modification required for embryonic development, transcriptional regulation, heterochromatin formation, X-inactivation, imprinting and genome stability [1–3]. The seminal DNA methylation inheritance model proposed nearly 40 years ago predicted the existence of enzymes that preferentially copy the parental DNA methylation pattern onto newly synthesized DNA strand in somatic cells and enzymes that set up patterns of DNA methylation in early embryonic development [4, 5]. Subsequent extensive studies have established the concept of DNMT1 as a maintenance methylation enzyme and DNMT3A and DNMT3B as *de novo* methylation enzymes [6–8]. However, growing evidence indicates that DNMT3A and DNMT3B also contribute to maintaining DNA methylation patterns in embryonic stem (ES) and somatic cells, as inactivation of these enzymes leads to gradual loss of DNA methylation, both at single copy genes and repetitive DNA sequences [8–12]. This is likely explained by the fact that DNMT1 is unable to replicate DNA methylation patterns with 100% accuracy [11, 12]. Thus, a current prevailing model suggests that the faithful inheritance of DNA methylation patterns in mammalian cells requires the coordinated functions of DNMT1 and DNMT3A/DNMT3B [13]. However, this working model raises a new question as to how the maintenance and *de novo* methylation activities are coordinated to permit the faithful inheritance of DNA methylation, because too much *de novo* methylation would tip the balance of DNA methylation inheritance to increased DNA methylation.

UHRF1 (also known as ICBP90 in human and NP95 in mouse) has emerged in recent years as a key epigenetic regulator essential for DNA maintenance methylation [14, 15]. As a multistructural and functional nuclear protein [14–19], UHRF1 associates DNA replication forks by binding hemimethylated CpG and methylated histone H3K9 (H3K9me2/3) [14, 15, 20, 21] and recruits DNMT1 through histone ubiquitination [22, 23]. Interestingly, although the mammalian genome also encodes a highly similar protein named UHRF2 (also known as NIRF), UHRF2 is neither required for DNA maintenance methylation nor able to substitute for UHRF1 in DNA maintenance methylation [24, 25].

The most common epigenetic alteration in cancer is global DNA hypomethylation [26–28]. DNA hypomethylation in cancer is generally observed in highly repetitive sequences including centromeric satellites, Alu and long interspersed elements (LINE1) that comprise approximately half of the genome. DNA hypomethylation can be a causal factor for tumorigenesis, as demonstrated by studies of DNMT-deficient mice [29, 30]. Conditional deletion of DNMT3A in mice promotes growth and progression, but not initiation, of lung tumor [31] and leads to global hypomethylation in lung cancer [32]. Furthermore, recurrent somatic DNMT3A mutations have been identified in acute myeloid leukemia and other hematological malignancies [33–35], indicating that impaired activity of DNMT3A is a causal factor of tumorigenesis. However, DNMT3B appears to function as an oncogene, as its deletion and overexpression have been shown to suppress and promote lung cancer, respectively [36, 37]. Nevertheless the mechanisms underlying the widespread DNA hypomethylation in cancer remain elusive.

In this study we uncover that both UHRF1 and UHRF2 are negative regulators of DNA *de novo* methylation by DNMT3A. Mechanistically, UHRF1 and UHRF2 inhibit *de novo* methylation by DNMT3A by functioning as E3 ligases promoting DNMT3A ubiquitination and degradation. Significantly, by analyzing the unrestricted paired tumor and normal control RNA-seq data available in the TCGA database, UHRF1 and to a less extent UHRF2 are found to be substantially overexpressed in all types of cancers. We present evidence that UHRF1/2 overexpression is likely a common mechanism for suppressing DNMT3A activity and consequently widespread DNA hypomethylation in cancers. We also propose a new DNA methylation inheritance model in which UHRF1/2 controls the fidelity of DNA methylation inheritance by coupling DNA maintenance methylation with inhibition of DNMT3A *de novo* methylation.

## Results

### UHRF2 negatively regulates DNA methylation in various cancer cell lines

**Figure 1:**
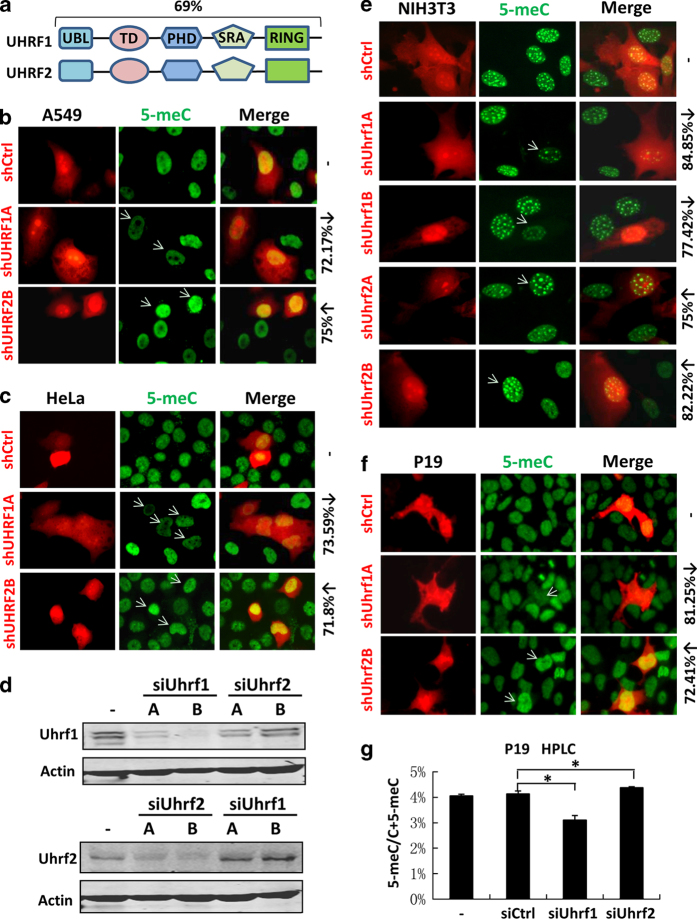
UHRF2 negatively regulates DNA methylation in both human and mouse cells. (**a**) Diagram illustrating the structural organization of human UHRF1 and UHRF2. PHD, plant homeodomain; RING, really interesting new gene domain; SRA, SET and ring-associated domain; TD, tandem TUDOR domain; UBL, ubiquitin-like domain. (**b**, **c**) Immunostaining analysis of 5-meC upon knockdown of UHRF1 or UHRF2 in A549 cells (**b**) and HeLa cells (**c**). Also shown is the percentage of cells with reduced (↓) DNA methylation in shUHRF1-transfected cells or increased (↑) DNA methylation in shUHRF2-transfected cells. The same labeling applied to immunostaining data in other figures. The shRNA vector was tagged with *RFP*. (**d**) Western blot analysis showing specific knockdown of Uhrf1 or Uhrf2 in P19 cells by siRNAs. (**e**, **f**) Immunostaining analysis of 5-meC upon knockdown of Uhrf1 or Uhrf2 in mouse NIH3T3 fibroblast cells (**e**) and P19 embryonic carcinoma cells (**f**). (**g**) Quantitative measurement of the levels of 5-meC in genomic DNA from P19 cells treated without or with control siRNA, siUhrf1 and siUhrf2. Asterisk indicates a *P*-value of <0.05.

Despite its similarity to UHRF1 in amino acid sequences and domain organization (Figure 1a), we and others have previously shown that UHRF2 is not required and cannot substitute UHRF1 for its role in DNA maintenance methylation [24, 25]. To examine if UHRF2 plays a role in DNA methylation, we knocked down UHRF2 in the human lung cancer cell line A549 using shRNA and examined the DNA methylation status by immunofluorescent staining using an anti-5-meC antibody. As a control, cells transfected with shUHRF1 exhibited reduced levels of DNA methylation (Figure 1b), in agreement with that UHRF1 is required for DNA maintenance methylation catalyzed by DNMT1 [14, 15]. In contrast, the cells transfected with shUHRF2 showed increased levels of DNA methylation (Figure 1b). The specificity of the inhibitory effect of the shRNAs against UHRF1 and UHRF2 was confirmed by western blot analysis (Supplementary Figure S1A). Essentially the same results were observed in HeLa cells (Figure 1c) as well as other cancer cell lines including SPC-A1, H460 and PC-9 (Supplementary Figure S1B). Together, these results suggest that, unlike UHRF1, UHRF2 negatively regulates DNA methylation in various human cancer cell lines.

### Uhrf2 negatively regulates DNA methylation in mouse embryonic carcinoma P19 and somatic cells

Orthologs of human UHRF2 have been identified in all mammals but not in organisms such as zebrafish. Thus, we wished to test if the negative regulation of DNA methylation is a conserved function of UHRF2. We designed and validated the efficacy and specificity of two small interfering RNAs (siRNAs) against each of the mouse Uhrf1 and Uhrf2 using mouse P19 embryonic carcinoma cells (Figure 1d). We also generated shRNA constructs based on these validated siRNA sequences. Consistent with the results from human cancer cell lines, transfection of mouse NIH3T3 fibroblast cells with both shUhrf1 constructs led to decreased levels of DNA methylation as revealed by immunofluorescent staining, whereas transfection of NIH3T3 cells with both shUhrf2 led to increased levels of DNA methylation (Figure 1e). Similar results were observed in P19 cells (Figure 1f) and mouse primary embryonic fibroblasts (MEF) (Supplementary Figure S1C). To validate these immunofluorescent staining-based results, we prepared genomic DNA from the P19 cells transfected with control siRNA, siUhrf1B and siUhrf2B, respectively, and quantitatively measured the levels of 5-meC in genomic DNA by high-performance liquid chromatography (HPLC) analysis. The results from three independent experiments confirmed an increased level of 5-meC in total C (from 4.1 to 4.35%, equivalent to a 6.1% increase in CpG methylation) in siUhrf2-treated cells and a reduced level of 5-meC (from 4.1 to 3.1%, equivalent to a 24% decrease in CpG methylation) in siUhrf1-treated cells (Figure 1g). Together, these results indicate that negative regulation of DNA methylation is a general and conserved function for UHRF2.

### UHRF2 inhibits DNA methylation independent of UHRF1

**Figure 2:**
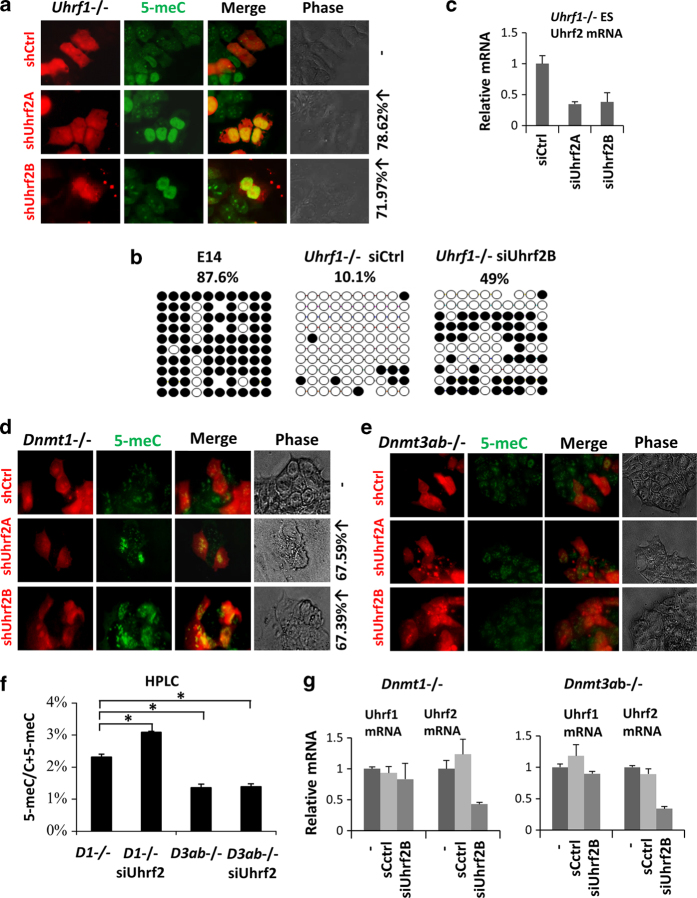
UHRF2 inhibits DNA methylation through negative regulation of *de novo* DNA methylation. (**a**) Immunostaining analysis of 5-meC for *Uhrf1*^−/−^ ES cells treated with control shRNA or shRNAs against Uhrf2. (**b**) Bisulfite sequencing analysis of DNA methylation in *IAP* repetitive sequences from control E14 and *Uhrf1*^−/−^ ES cells treated with control siRNA or siUhrf2B. (**c**) qRT-PCR analysis showing efficient downregulation of Uhrf2 mRNA upon treatment of *Uhrf1*^−/−^ ES cells with two different siUhrf2A and siUhrf2B. Bars and error bars represent mean values and s.e.m. from three measurements, respectively. (**d**) Immunostaining analysis of 5-meC for *Dnmt1*^−/−^ ES cells treated with control shRNA or shRNAs against Uhrf2. (**e**) Immunostaining analysis of 5-meC for *Dnmt3ab*^−/−^ ES cells treated with control shRNA or shRNAs against Uhrf2. (**f**) Quantitative measurement of the levels of 5-meC of genomic DNA from *Dnmt1*^−/−^ ES cells (D1^−/−^) or *Dnmt3ab*^−/−^ (D3ab^−/−^) treated without or with siUhrf2 by HPLC analysis. Asterisk indicates a *P*-value of <0.05. (**g**) qRT-PCR analysis showing efficient downregulation of Uhrf2 mRNA upon treatment of *Dnmt1*^−/−^ ES cells or *Dnmt3a/3b*^−/−^ ES cells with siUhrf2B. Bars and error bars represent mean values and s.e.m. from three measurements, respectively.

Next we wished to determine the underlying mechanism by which UHRF2 negatively regulates DNA methylation. Like UHRF1, UHRF2 also binds hemimethylated DNA and H3K9me2/3 [24], raising the possibility that UHRF2 may negatively regulate DNA methylation by inhibiting UHRF1-mediated maintenance methylation. If this were the case, the inhibitory effect of Uhrf2 would not be observed in the *Uhrf1*^−/−^ mouse ES cells. We tested this by transfecting *Uhrf1*^−/−^ ES cells with two different shRNAs against Uhrf2 and analyzing the DNA methylation status by immunofluorecent staining. Representative results in Figure 2a demonstrated that transfection of *Uhrf1*^−/−^ ES cells with both shUhrf2 all led to increased DNA methylation. In addition, we knocked down Uhrf2 in *Uhrf1*^−/−^ ES cells using two different siRNAs and examined the effect on DNA methylation by bisulfite sequencing and immunofluorecent staining. Bisulfite sequencing analysis demonstrated that knockdown of Uhrf2 by siRNA resulted in increased methylation of the repetitive sequences IAP (from 10.1 to 49%) (Figure 2b). In addition, immunofluorecent staining analysis confirmed increased DNA methylation in siRNA-treated *Uhrf1*^−/−^ ES cells (Supplementary Figure S2). The knockdown of Uhrf2 in siRNA-treated *Uhrf1*^−/−^ ES cells was verified by quantitative reverse transcription-PCR (qRT-PCR) analysis (Figure 2c). Together these results indicate that Uhrf2 negatively regulates DNA methylation independently of Uhrf1.

### Uhrf2 inhibits DNA methylation through inhibition of *de novo* DNA methylation

We next wished to determine whether UHRF2 inhibits maintenance methylation by DNMT1 or *de novo* methylation by DNMT3A and/or DNMT3B. To this end, we transfected *Dnmt1* knockout mouse ES cells (*Dnmt1*^−/−^) and *Dnmt3a* and *Dnmt3b* double knockout mouse ES cells (*Dnmt3ab*^−/−^) with shRNAs against Uhrf2. The subsequent immunofluorescent analysis for 5-meC revealed increased levels of DNA methylation in *Dnmt1*^−/−^ ES cells transfected with either of the two shUhrf2 plasmids (Figure 2d). In contrast, no increase of DNA methylation was observed in the shUhrf2-transfected *Dnmt3ab*^−/−^ ES cells (Figure 2e). Similarly, immunofluorescent analysis of 5-meC showed that knockdown of Uhrf2 by siRNA against Uhrf2 led to increased DNA methylation in *Dnmt1*^−/−^ ES cells, but not in *Dnmt3ab*^−/−^ ES cells (Supplementary Figure S3). We also quantitatively analyzed the levels of DNA methylation in genomic DNA samples derived from both types of ES cells treated with control siRNA or siUhrf2B. The representative results in Figure 2f show that siUhrf2B treatment led to increased 5-meC in *Dnmt1*^−/−^ ES cells (from ~2.4 to ~3.2% in comparison with control siRNA-treated cells), but not in *Dnmt3ab*^−/−^ ES cells. The knockdown of Uhrf2 mRNA was verified by RT-PCR analysis (Figure 2g). Together, these results indicate that Uhrf2 inhibits *de novo* methylation by Dnmt3a and/or Dnmt3b, but not the maintenance methylation by Dnmt1, consistent with the results described above that Uhrf2 inhibits DNA methylation independently of Uhrf1, a protein essential for maintenance methylation by Dnmt1.

### Both UHRF2 and UHRF1 negatively regulate DNMT3A at the level of protein but not transcription

**Figure 3:**
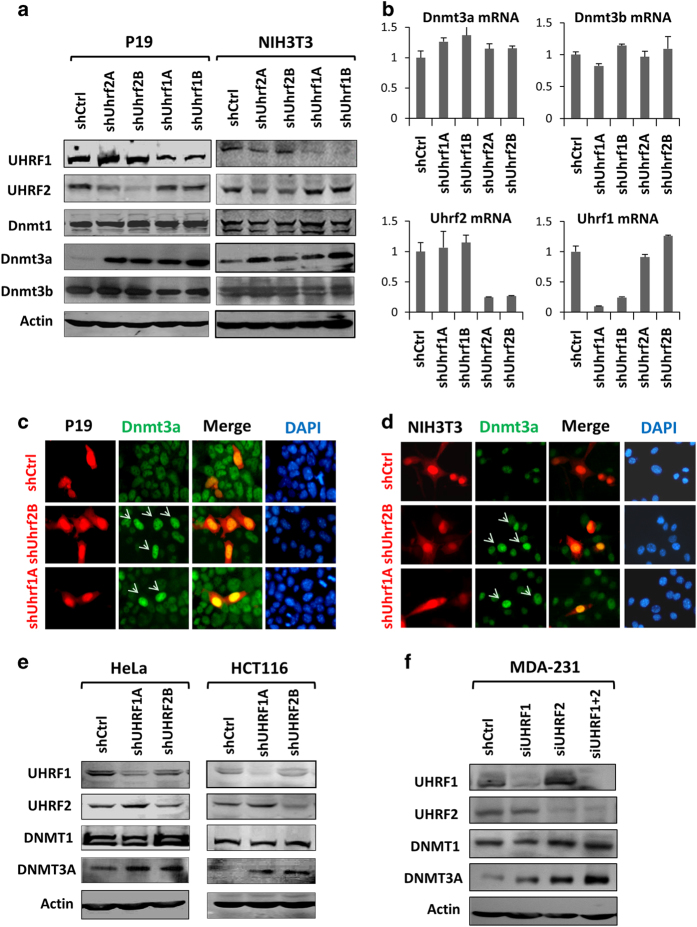
Both UHRF2 and UHRF1 negatively regulate DNMT3A post-transcriptionally in mouse and human cancer cells. (**a**) Western blot analysis of Dnmt1, Dnmt3a and Dnmt3b upon knockdown of Uhrf1 or Uhrf2 in mouse P19 and NIH3T3 cells. Knockdown of Uhrf1 or Uhrf2 was achieved by lentiviral-mediated shRNA infection. (**b**) qRT-PCR analysis of the levels of Dnmt3a and Dnmt3b mRNAs in shUhrf1- or shUhrf2-infected P19 cells. The knockdown of Uhrf1 or Uhrf2 was verified by qRT-PCR analysis. Bars and error bars represent mean values and s.e.m. from three measurements, respectively. (**c**, **d**) Immunostaining analysis of Dnmt3a proteins for P19 cells (**c**) and NIH3T3 cells (**d**) transfected with control shRNA vector or shRNA against Uhrf1 or Uhrf2. Arrows indicate the cells transfected with either shUhrf2B or shUHRF1A. (**e**) Western blot analysis of DNMT1 and DNMT3A in whole-cell extracts derived from HeLa and HCT116 infected with lentiviruses encoding shUHRF1A or shUHRF2B. (**f**) Western blot analysis of DNMT1 and DNMT3A in breast cancer cell line MDA-231 infected with lentiviruses encoding shUHRF1A or shUHRF2B or both. Note the double knockdown of UHRF1 and UHRF2 resulted in a more pronounced increase of DNMT3A than knockdown of either one.

Having established that Uhrf2 inhibits DNA methylation in a Dnmt3a and/or Dnmt3b-dependent manner in mouse ES cells, we next examined whether Uhrf2 inhibits the expression, protein stability or enzymatic activity of Dnmt3a and/or Dnmt3b. We knocked down Uhrf2 in mouse P19 cells using lentiviruses encoding control or shRNAs against Uhrf1 or Uhrf2 and subsequently examined the levels of proteins and mRNAs of Dnmt3a and Dnmt3b by western blot and RT-PCR analysis, respectively. We observed that knockdown of Uhrf2 in P19 cells led to substantially increased proteins of Dnmt3a but not Dnmt3b (Figure 3a). However, RT-PCR analysis revealed no significant changes of either *Dnmt3a* or *Dnmt3b* mRNA (Figure 3b). By the same approach, we observed that knockdown of Uhrf2 in NIH3T3 cells led to increased Dnmt3a but not Dnmt3b at the level of proteins (Figure 3a) but not mRNA (data not shown). These data suggest that Uhrf2 inhibits DNA methylation by negative regulation of Dnmt3a through a post-transcriptional mechanism.

Initially attempted as controls, we also knocked down Uhrf1 in P19 cells with shRNAs. We were surprised to observe that this also led to a substantial increase of Dnmt3a proteins (Figure 3a), despite the observed opposite effect on DNA methylation upon knockdown of Uhrf1 and Uhrf2 (Figure 1). The specific knockdown of Uhrf2 or Uhrf1 by their respective shRNAs was confirmed by western blot analysis (Figure 3a). Again RT-PCR analysis showed that knockdown of Uhrf1 had no effect on the levels of mRNAs of Dnmt3b or Dnmt3a in P19 cells (Figure 3b). Similarly, knockdown of Uhrf1 in NIH3T3 cells led to increased levels of Dnmt3a but not Dnmt1 and Dnmt3b proteins (Figure 3a).

As an independent assay for detecting increased levels of Dnmt3a proteins, we transfected P19 and NIH3T3 cells with control shRNA or shRNAs against either Uhrf2 or Uhrf1 and assayed the Dnmt3a protein level by immunofluorescent staining. Figure 3c and d showed that the cells transfected with either shUhrf2B or shUhrf1A exhibited increased Dnmt3a proteins. Similarly, knockdown of either Uhrf1 or Uhrf2 in mouse R1 ES cells or primary MEFs resulted in increased Dnmt3a proteins (Supplementary Figure S4). Together, these experiments with mouse cell lines indicated that both Uhrf2 and Uhrf1 negatively regulate Dnmt3a expression at the level of protein but not transcription.

To determine whether UHRF2 and UHRF1 also negatively regulate DNMT3A in human cancer cell lines, we knocked down UHRF2 or UHRF1 in cervical cancer HeLa and colorectal cancer HCT116 cells by lentiviral-mediated shRNA infection. Subsequent western blot analyses showed that knockdown of either UHRF2 or UHRF1 increased the levels of DNMT3A but not DNMT1 (Figure 3e). RT-PCR analysis showed that knockdown of UHRF2 did not significantly affect the transcription of DNMT3A (data not shown). Under the same conditions we were unable to consistently detect DNMT3B by western blot using several commercially available antibodies (data not shown), suggesting that DNMT3B is in relative low abundance in these cell lines. Furthermore, while knockdown of either UHRF1 or UHRF2 led to increased DNMT3A, a more pronounced increase of DNMT3A was observed when both UHRF1 and UHRF2 were knocked down in the MDA-231 breast cancer cell line (Figure 3f), indicating that UHRF1 and UHRF2 cooperatively regulate DNMT3A. Taken together, these results suggest that UHRF2 negatively controls DNMT3A but not DNMT3B at the level of protein but not transcription, thereby providing a mechanism by which UHRF2 negatively regulates DNA methylation. The finding that UHRF1 also negatively regulates DNMT3A suggests that UHRF1, in addition to its essential role in DNA maintenance methylation, may also have a role in negative regulation of *de novo* methylation by DNMT3A.

### Both UHRF2 and UHRF1 induce DNMT3A degradation by acting as ubiquitin E3 ligases

**Figure 4:**
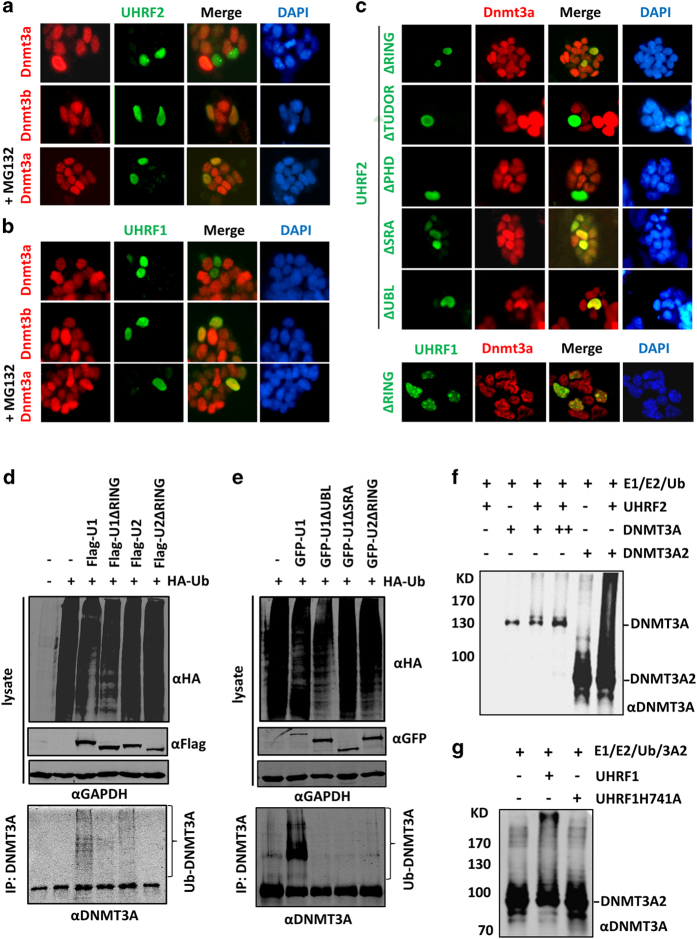
Both UHRF1 and UHRF2 promote DNMT3A ubiquitination and proteasome-dependent degradation. (**a**, **b**) Overexpression of UHRF2 or UHRF1 in mouse R1 ES cells induced Dnmt3a but not Dnmt3b degradation. The R1 ES cells were transfected with Flag-UHRF2 (**a**) or Flag-UHRF1 (**b**) and the levels of Dnmt3a or Dnmt3b were analyzed by immunostaining. MG132 (20 μm) was added 6 h before immunostaining. (**c**) Mapping the functional domains required for UHRF1/2-mediated Dnmt3a degradation. Flag-UHRF2 with deletion of each individual domain as indicated was transfected into R1 ES cells and the effect on Dnmt3a was analyzed by immunostaining. Also shown is the immunostaining for Flag-UHRF1 with deletion of RING domain. (**d**) UHRF1 and UHRF2 promoted endogenous DNMT3A ubiquitination in 293T cells in a RING domain-dependent manner. Flag-tagged UHRF1 or UHRF2 or ∆RING mutant was co-transfected with 6xHis-Ub into 293T cells. The DNMT3A ubiquitination was detected by western blot using anti-DNMT3A antibody. (**e**) The experiments were essentially as in (**d**) except various UHRF1 deletion mutants were used. (**f**) Recombinant UHRF2 catalyzed DNMT3A and a short form DNMT3A2 ubiquitination in *in vitro* ubiquitination assay. (**g**) UHRF1 promoted DNMT3A ubiquitination *in vitro* in an E3-ligase activity-dependent manner. The UHRF1 H741A mutant is inactive in E3 ligase activity (Supplementary Figure S6).

Both UHRF2 and UHRF1 are RING domain-containing proteins with E3 ubiquitin ligase activity [38–40]. Thus, we next investigated whether UHRF2 and UHRF1 regulate DNMT3A protein stability through ubiquitin-dependent proteasome degradation. Flag-tagged human UHRF2 or UHRF1 were expressed in mouse R1 ES cells that contain a relatively high level of endogenous Dnmt3a and the effect on Dnmt3a was investigated by immunofluorescent staining. Results in Figure 4a showed that expression of Flag-UHRF2 substantially reduced the levels of Dnmt3a but did not affect the levels of Dnmt3b (Figure 4a). Addition of MG132, a proteasome inhibitor, 6 h before immunofluorescent staining effectively blocked the UHRF2-induced reduction of Dnmt3a (Figure 4a), suggesting that UHRF2 downregulated Dnmt3a through proteasome degradation. Similarly, we found that expression of Flag-UHRF1 also resulted in reduced Dnmt3a but not Dnmt3b in R1 ES cells, and this effect was blocked by the addition of MG132 (Figure 4b).

We next examined if the RING domain is required for UHRF1/2-mediated degradation of DNMT3A. We found that deletion of the RING finger domain (ΔRING) from UHRF2 abolished its ability to downregulate DNMT3A in R1 ES cells (Figure 4c). Similarly, deletion of the RING finger domain from Flag-UHRF1 also abolished its ability to induce DNMT3A degradation in R1 ES cells (Figure 4c). In further support for a role of the UHRF1/2 E3 ubiquitin ligase in DNMT3A degradation, a UHRF1 mutant impaired in E3 ligase activity due to change of three cysteine residues in RING domain to alanine (3C/3A) was unable to induce DNMT3A degradation in R1 ES cells (Supplementary Figure S5A). Together these data suggest that UHRF1/2 targets DNMT3A most likely through its RING domain-dependent E3 ligase activity.

Using a panel of deletion mutants, we also examined the potential roles of other UHRF2 structural domains in targeting Dnmt3a degradation. The results in Figure 4c showed that the deletion of either PHD or TUDOR domain from UHRF2 had no effect, whereas deletion of either the UBL or SRA domain impaired the ability of UHRF2 to induce Dnmt3a degradation (Figure 4c). The requirement for the UBL domain could be explained by our co-immunoprecipitation assay showing that the UBL domain of UHRF2 is required for interaction with DNMT3A (Supplementary Figure S5B). However, deletion of SRA did not significantly affect the UHRF2–DNMT3A interaction (Supplementary Figure S5B). As endogenous Dnmt3a proteins were essentially all associated with chromatin 41, we surmised that the SRA domain could be required for UHRF1/2 to associate with chromatin and subsequently promote Dnmt3a degradation. Consistent with this idea, we found that the UHRF1 SRA mutants with impaired hemi-mCpG binding activity were unable to induce Dnmt3a degradation, whereas a mutant with impaired H3K9me2/3-binding activity maintained the DNMT3A degradation activity (Supplementary Figure S5C).

We next tested if UHRF1/2 promoted DNMT3A ubiquitination. To this end, we first tested the ability of UHRF1 and UHRF2 as well as their deletion of RING mutants to promote ubiquitination of endogenous DNMT3A in 293T cells. Figure 4d showed that both wild-type UHRF1 and UHRF2 promoted DNMT3A ubiquitination, whereas the RING deletion mutants failed to do so. Consistent with the above data that both the UBL and SRA domains were required for UHRF2-induced DNMT3A degradation, the UBL and SRA deletion mutants also failed to promote the ubiquitination of endogenous DNMT3A in 293T cells (Figure 4e). To test if UHRF1 and UHRF2 are authentic DNMT3A ubiquitin E3 ligases, we prepared recombinant GST-tagged UHRF1 and UHRF2 from bacteria and 6xHis-tagged recombinant DNMT3A and DNMT3A2 from insect cells. Using auto-ubiquitination as an assay for E3 ligase activity, we found that the UHRF1 E3 ligase activity is dependent on RING but not SRA domain (Supplementary Figure S6). Using this *in vitro* reconstituted ubiquitin assay system, we found that UHRF2 was able to catalyze DNMT3A and DNMT3A2 ubiquitination (Figure 4f). Furthermore, the ability of UHRF1 to catalyze DNMT3A2 ubiquitination was dependent on its E3 ligase activity, because recombinant UHRF1 H741A, a mutant that was impaired in E3 ligase activity in cellular based assay, failed to catalyze DNMT3A2 ubiquitination in our *in vitro* assay (Figure 4g). Together, these results indicate that UHRF2 and UHRF1 function as E3 ligases by catalyzing DNMT3A ubiquitination and their E3 ligase activity is dependent on the RING domain.

### UHRF1 also negatively regulates *de novo* DNA methylation

**Figure 5:**
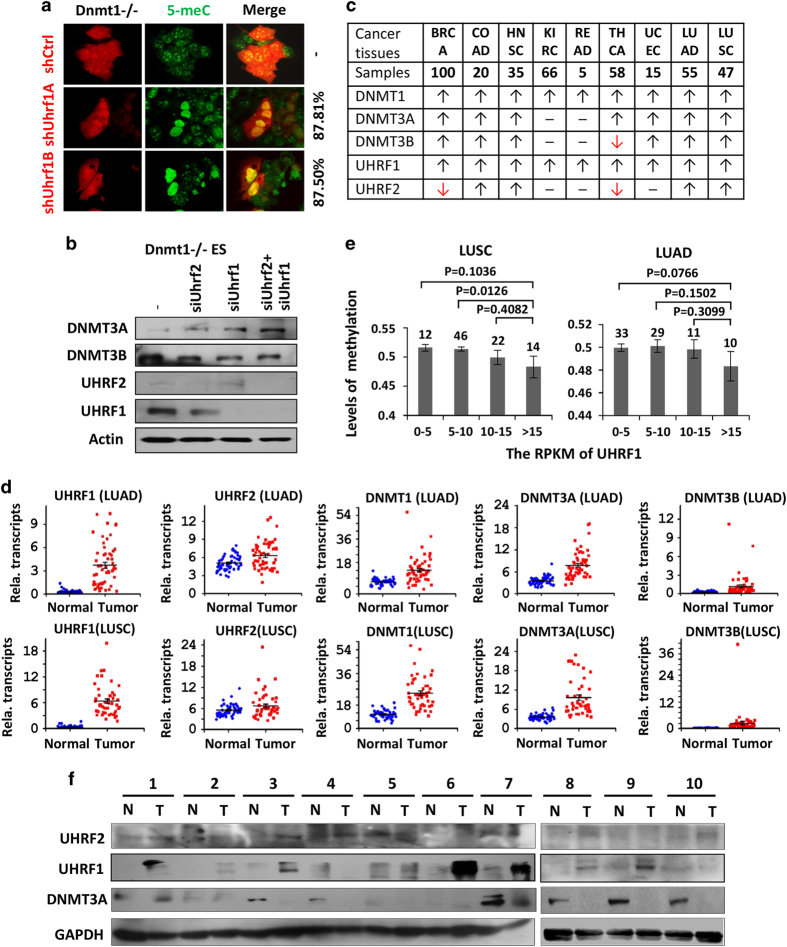
UHRF1 also negatively regulates *de novo* DNA methylation and UHRF1/2 overexpression in cancers correlates with downregulation of DNMT3A proteins. (**a**) Immunostaining analysis of 5-meC in *Dnmt1*^−/−^ ES cells transfected with two different shRNAs against Uhrf1. Increased 5-meC staining was observed for cells transfected with shUhrf1A or shUhrf1B. (**b**) Western blot analysis of Dnmt3a and Dnmt3b in *Dnmt1*^−/−^ ES cells treated with siRNA against either Uhrf1 (siUhrf1B) or Uhrf2 (siUhrf2B) or both. (**c**) Summary of RNA-seq data from the TCGA database for eight different types of cancers. The numbers of paired control and tumor samples for each type of cancers are indicated. ↑, upregulated; ↓, downregulated; –, no significant change. (**d**) The relative levels of UHRF1, UHRF2, DNMT1, DNMT3A and DNMT3B transcripts in controls and tumors for lung adenocarcinoma (LUAD) and lung squamous cell carcinoma (LUSC). Note both UHRF1/2 and DNMT3A are overexpressed in these cancers. (**e**) The relationship between UHRF1 overexpression and the levels of DNA methylation in LUSC and LUAD based on TCGA RNA-seq and Illumina 450K methylation array data. The number of tumor samples was shown at the top of each bar. The *P*-value for each group of tumor samples with the samples with UHRF1 RPKM>15 was also shown at the top. (**f**) Western blot analysis of paired normal control and tumor samples from 10 lung cancer patients. N, tumor adjacent normal control; T, tumor. Note downregulation of DNMT3A proteins in tumor samples.

The above results demonstrated that UHRF1 also negatively regulates DNMT3A by inducing its degradation. To test if UHRF1, besides its essential role in DNA maintenance methylation, also negatively regulates DNA *de novo* methylation, we resorted to *Dnmt1*^−/−^ ES cells in which *de novo* methylation by Dnmt3a is intact while maintenance methylation is abolished. We transfected the *Dnmt1*^−/−^ ES cells with shUhrf1 and analyzed the effect on DNA methylation by immunofluorescent staining. Remarkably, we observed significantly increased levels of DNA methylation in shUhrf1-transfected *Dnmt1*^−/−^ ES cells (Figure 5a), much like in the *Dnmt1*^−/−^ ES cells transfected with shUhrf2 (Figure 2d). Thus, like Uhrf2, Uhrf1 also negatively regulates *de novo* DNA methylation, presumably through negative regulation of Dnmt3a. In support of this idea, knockdown of either Uhrf2 or Uhrf1 in *Dnmt1*^−/−^ ES cells resulted in increased levels of Dnmt3a, but simultaneous knockdown of both Uhrf1 and Uhrf2 led to a more pronounced increase of Dnmt3a proteins (Figure 5b), indicating that Uhrf1 and Uhrf2 cooperatively target Dnmt3a for degradation. Consistent with the idea that UHRF1/2 negatively regulates *de novo* methylation by targeting DNMT3A degradation, we found that addition of UHRF1 or UHRF2 did not inhibit the DNA methylation activity of DNMT3A *in vitro* (Supplementary Figure S7). In addition, *de novo* methylation of a reporter plasmid in cells by DNMT3A was also not inhibited by ectopically expressed UHRF1 or UHRF2 when the levels of DNMT3A proteins were kept relatively constant with ectopically expressed DNMT3A (Supplementary Figure S8). Finally, we tested if changing the levels of DNMT3A were sufficient to alter global DNA methylation in cells. We found that knockdown of Dnmt3a in R1 ES cells was sufficient to downregulate the global DNA methylation in R1 ES cells, whereas ectopic expression of DNMT3A in primary MEF cells was sufficient to upregulate the global DNA methylation in transfected MEF cells as shown by immunofluorescent staining assay (Supplementary Figure S9). We thus conclude that UHRF1/2 could downregulate global DNA methylation through their ability to induce DNMT3A degradation.

### UHRF1 is highly overexpressed in various cancers

Global DNA hypomethylation is a common epigenetic alteration of cancers. Furthermore, highly recurrent somatic DNMT3A mutations have been identified in several types of leukemia and are associated with poor prognosis of the diseases [33, 34], suggesting a causal link between loss of DNMT3A function and tumorigenesis. Having established that both UHRF1 and UHRF2 target DNMT3A for degradation and that downregulation of DNMT3A correlates with DNA hypomethylation, we next investigated the expression profiles of UHRF1 and UHRF2 in various cancers. In this regard, UHRF1 has been reported to be frequently overexpressed in cancers [17, 42–44]. By analyzing the comprehensive RNA-seq data for nine types of cancers that were derived from the paired normal controls and tumors and were unrestrictedly available in the TCGA database (http://cancergenome.nih.gov/), we found that UHRF1 is universally overexpressed in all nine types of cancers (Figure 5c), whereas UHRF2 is overexpressed in some but not in all cancers (Figure 5c). Consistent with previous publications, these RNA-seq data indicate that both DNMT1 and DNMT3A are also frequently overexpressed in these cancers (Figure 5c). Figure 5d shows the relative levels of UHRF1, UHRF2, DNMT1, DNMT3A and DNMT3B transcripts in normal controls vs tumors for two types of lung cancers, lung adenocarcinoma and lung squamous cell carcinoma, and data for other types of cancers are presented in Supplementary Figure S10. We also analyzed the relationship between the levels of UHRF1 expression and the levels of DNA methylation based on RNA-seq and Illumina 450K methylation array data available for lung squamous cell carcinoma and lung adenocarcinoma in the TCGA database. We found that increased UHRF1 expression indeed correlated with increased DNA hypomethylation in these samples, more evidently for lung squamous cell carcinoma (Figure 5e). Thus, current RNA-seq data provide compelling evidence that UHRF1 and to less extent UHRF2 are overexpressed in various cancers and its overexpression appears to correlate with DNA hypomethylation phenotype in cancer, raising the possibility that UHRF1/2 overexpression would downregulate DNMT3A proteins and consequently lead to DNA hypomethylation phenotypes in cancers.

### UHRF1/2 overexpression correlates with substantially reduced DNMT3A proteins in lung cancers

To test if UHRF1/2 overexpression indeed leads to downregulation of DNMT3A proteins in cancers despite DNMT3A overexpression at the level of transcription (Figure 5c), we next examined the levels of UHRF1, UHRF2 and DNMT3A proteins in paired lung tumors and adjacent normal controls by western blot analysis. Although UHRF2 was expressed at a relatively low level in both tumor samples and controls, increased UHRF2 proteins could be detected in 4 out of 10 tumor samples (Figure 5f). Consistent with RNA-seq data showing UHRF1 overexpression in lung cancers, the increased UHRF1 proteins could be clearly detected in 7 out of 10 tumor samples (Figure 5f). Significantly, although RNA-seq data indicate increased DNMT3A transcripts in lung cancers, substantially reduced levels of DNMT3A proteins were actually detected in the tumors, and the levels of DNMT3A proteins exhibited a strong inverse correlation with that of the increased UHRF1 and UHRF2 (Figure 5f). Thus, despite increased DNMT3A transcripts in lung cancers, DNMT3A at the level of proteins is actually substantially reduced in lung tumors, most likely as a consequence of targeted degradation of DNMT3A by overexpressed UHRF1/2.

### UHRF1/2 negatively regulates DNMT3A proteins in multiple lung cancer cell lines

**Figure 6:**
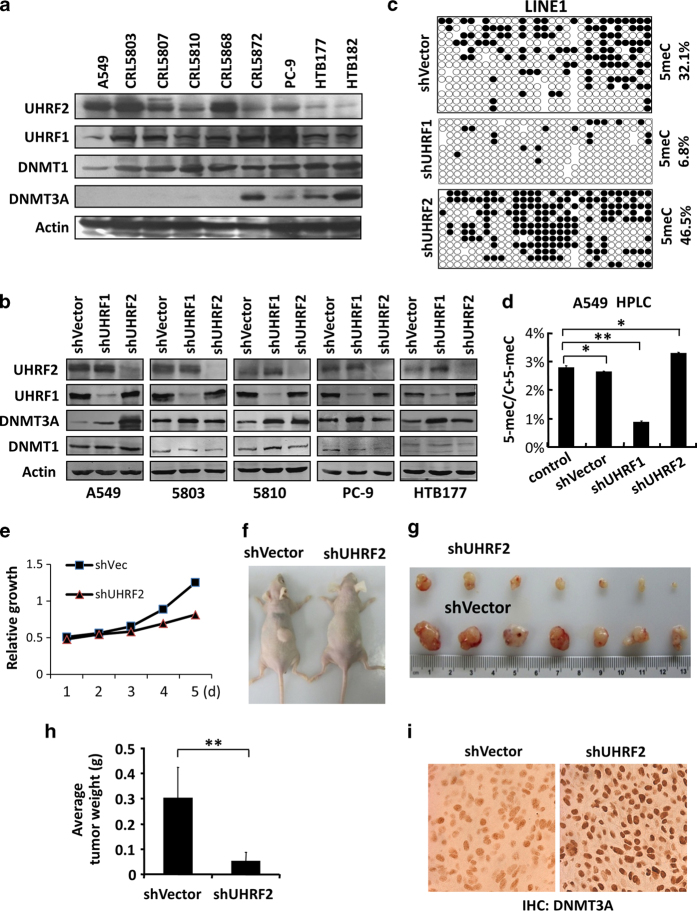
Negative regulation of DNMT3A by overexpressed UHRF1/2 contributes to DNA hypomethylation in cancers and promotes tumor growth. (**a**) Western blot analysis of a panel of lung cancer cell lines showing a general inverse correlation between UHRF1/2 and DNMT3A at the level of proteins. (**b**) Western blot analysis showing that UHRF1 and UHRF2 negatively regulate DNMT3A in lung cancer cell lines. UHRF1 or UHRF2 was knocked down in five lung cancer cell lines by lentiviral-mediated shRNA infection. (**c**) Bisulfite sequencing analysis of DNA methylation status of the LINE1 sequence in DNA from control, shUHRF1- and shUHRF2-expressing A549 cells. (**d**) Quantitative measurement of DNA methylation in A549 cells with knockdown of either UHRF1 or UHRF2 by HPLC. Asterisk indicates a *P*-value of <0.05 and double asterisk indicates a *P*-value of <0.01. (**e**) The comparison of cell proliferation between A549 cells with or without knockdown of UHRF2. The relative proliferation was determined with the starting cell number 0.5×10^5^ at 0.5. (**f**) The representative image of tumor growth for 6 weeks in mice injected subcutaneously with control and shUHRF2-expressing A549 cells. (**g**) The image of tumors recovered from the mice injected subcutaneously with control and shUHRF2-expressing A549 cells 6 weeks after injection. (**h**) The average weights of the tumors derived from control and shUHRF2-expressing A549 cells. A Student’s *t*-test was used to calculate the statistical significance. Double asterisk indicates a *P*-value of <0.01. (**i**) Immunohistochemistry verified the elevated levels of DNMT3A in tumors derived from shUHRF2-expressing A549 cells.

Having observed an inverse correlation between UHRF1/2 and DNMT3A proteins in lung cancer specimens, we next examined further the causal role of UHRF1/2 overexpression in downregulation of DNMT3A proteins in multiple lung cancer cell lines. Western blot analysis in Figure 6a revealed a general inverse correlation between the levels of UHRF1 and UHRF2 and that of DNMT3A in these cell lines, whereas DNMT1 was more uniformly detected. Furthermore, as shown in Figure 6b, knockdown of UHRF2 led to a substantial increase of DNMT3A in A549 cells, whereas the increase of DNMT3A upon knockdown of UHRF2 was more subtle in CRL5803, CRL5810, PC-9 and HTB177 cell lines. On the other hand, knockdown of UHRF1 led to increased DNMT3A (2- to 4-fold) not only in A549 cells, but also in all other four cell lines. As a comparison, knockdown of UHRF1 only led to a moderate increase of DNMT1 in CRL5810 (Figure 6b) but not in other cell lines. These data indicate that overexpression of UHRF1/2 causally downregulates DNMT3A proteins in cancer cells. Also in agreement with the observed widespread overexpression of UHRF1 in cancers, our data suggest that UHRF1 is likely to have a broader role in negative regulation of DNMT3A than UHRF2.

### Negative regulation of DNMT3A by UHRF1/2 as a mechanism for DNA hypomethylation in cancer

We next wished to test whether negative regulation of DNMT3A by UHRF1/2 contributes to DNA hypomethylation in cancer. As UHRF1 is also required for DNA maintenance methylation, we could not manipulate the level of UHRF1 in cancer cell lines to test whether negative regulation of DNMT3A by UHRF1 contributes to DNA hypomethylation in cancer. However, as UHRF2 is not required for DNA maintenance methylation, we resorted to A549 cells in which DNMT3A is negatively regulated primarily by UHRF2 (Figure 6b). We verified by immunofluorescent staining that transfection of A549 cells with two different shUHRF2 plasmids all led to substantially increased levels of DNMT3A proteins (Supplementary Figure S11A) and increased levels of DNA methylation (Supplementary Figure S11B). We then generated A549 cell lines stably expressing shVector, shUHRF1 and shUHRF2. The genomic DNA was prepared from these cell lines and subjected to analysis of DNA methylation in repetitive sequence LINE1 by bisulfite sequencing and global levels of DNA methylation by HPLC. As shown in Figure 6c, increased LINE1 methylation (from 32.1% in control to 46.5%) was observed in DNA from shUHRF2-expressing A549 cells. HPLC analysis also showed an increase of global DNA methylation in shUHRF2 A549 cells (from ~2.8% in control to ~3.3%) (Figure 6d). In contrast and as expected, knockdown of UHRF1 led to reduced methylation in LINE1 (Figure 6c) and reduced global levels of DNA methylation (Figure 6d). The substantial increase in LINE1 methylation in shUHRF2-expressing A549 cells is consistent with the notion that DNA hypomethylation in cancer occurs primarily in repetitive sequences and that DNMT3A plays a critical role in maintaining DNA methylation in repetitive sequences [8, 11, 32]. Thus, negative regulation of DNMT3A by overexpressed UHRF1/2 in cancers is likely to be a general mechanism for cancer-associated DNA hypomethylation.

### Increased DNMT3A and DNA methylation upon knockdown of UHRF2 impair tumor growth

We next investigated if negative regulation of DNMT3A by UHRF1/2 and its consequent DNA hypomethylation play a role in tumorigenesis. We noticed that shUHRF2 A549 cells exhibited a reduced growth rate compared with the shVector control A549 cells (Figure 6e). We then compared tumor growth for these two A549 cell lines in a nude mice xenograft model. When the same amounts of cells (5 millions) were injected subcutaneously into the back of nude mice, the shUHRF2-expressing A549 cells gave rise to tumors that were substantially smaller in volume than the control shRNA-expressing A549 cells (Figure 6f and g). By weight the tumors derived from the shUHRF2-expressing A549 cells were in average six to seven times smaller than those from the control shRNA-expressing A549 cells (Figure 6h). Immunohistochemistry staining for DNMT3A confirmed increased levels of DNMT3A in the tumors derived from the shUHRF2-expressing A549 cells (Figure 6i), indicating that increased DNMT3A levels upon downregulation of UHRF2 are associated with drastically reduced tumor growth.

### Overexpression of wild type but not mutant DNMT3A impairs tumor growth

**Figure 7:**
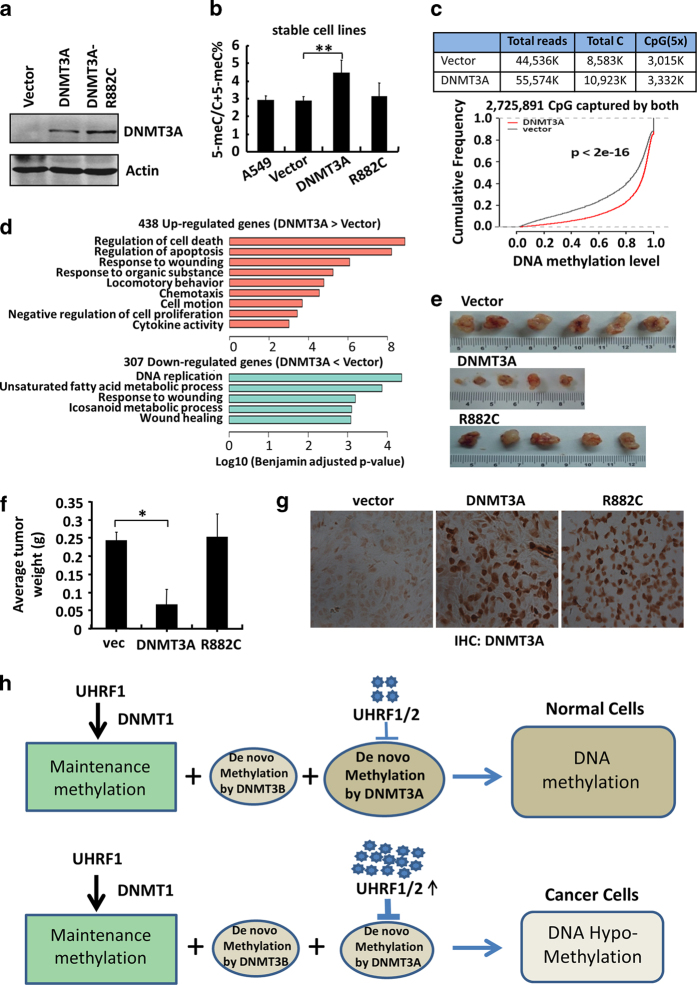
Overexpression of wild type but not enzymatic activity-deficient mutant DNMT3A increases DNA methylation and impairs tumor growth. (**a**) Western blot analysis of A549 cell lines expressing either the wild type or enzymatic deficient mutant DNMT3A. (**b**) Quantitative measurement of DNA methylation in A549 cells expressing either the wild type or enzymatic deficient mutant DNMT3A by HPLC. (**c**) Summary of RRBS analysis on DNMT3A expressing and control A549 cells. DNMT3A expression led to global increase of DNA methylation among commonly captured CpG sites. (**d**) The gene ontology analysis showing enriched functional annotation of differentially expressed genes between DNMT3A expressing and vector control A549 cell lines. The significance of enrichment was determined by DAVID. (**e**) The image of tumors recovered from the mice 6 weeks after injection subcutaneously with A549, A549-expressing DNMT3A and A549-expressing mutant DNMT3A. (**f**) The average weights of the tumors derived from A549, A549-expressing DNMT3A and A549-expressing mutant DNMT3A. A Student’s *t*-test was used to calculate the statistical significance. Asterisk indicates a *P*-value of <0.05. (**g**) Immunohistochemistry verified the sustained overexpression of either wild type or mutant DNMT3A in tumors derived from the corresponding injected A549 cells. (**h**) A working model for control of DNA methylation fidelity by the UHRF family proteins and overexpression of 
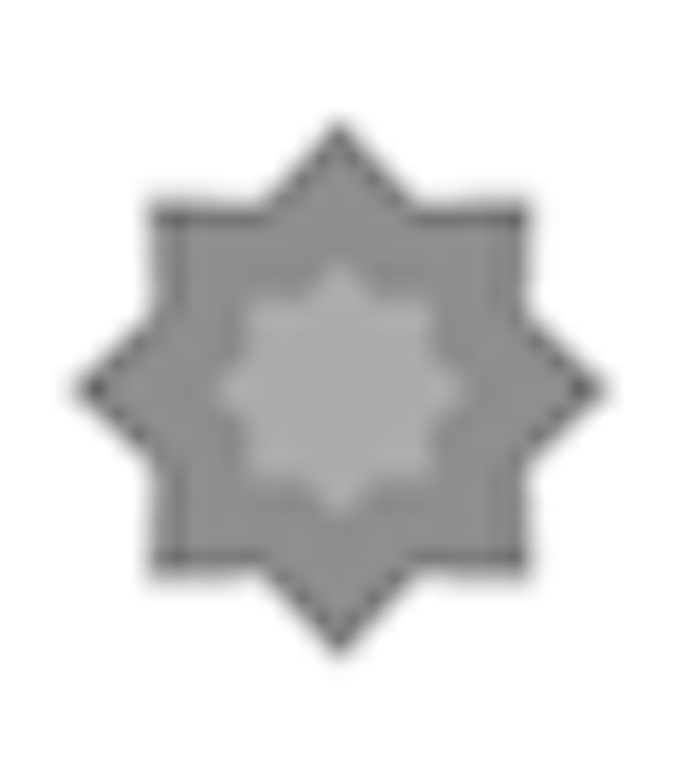
UHRF1 as a mechanism for widespread DNA hymomethylation in cancers. The faithful maintenance of patterns of DNA methylation through cell divisions is largely dependent on the activity of maintenance enzyme DNMT1 but also requires the activity of *de novo* enzymes DNMT3A and DNMT3B. However, too much of *de novo* methylation activity would lead to gradually increased DNA methylation and altered epigenetic regulation. The UHRF1 mediates maintenance methylation by targeting DNMT1 to replication forks. UHRF1/2 also keeps *de novo* activity under the check by targeting DNMT3A degradation. These dual functions for UHRF family proteins serve to safeguard the fidelity of DNA methylation inheritance in somatic cells, as shown in the upper panel. However, UHRF1/2 is highly and widely overexpressed in various cancers. Their overexpression leads to excessive degradation of DNMT3A, which in turn results in DNA hypomethylation and contributes to tumorigenesis, as illustrated in the lower panel. 
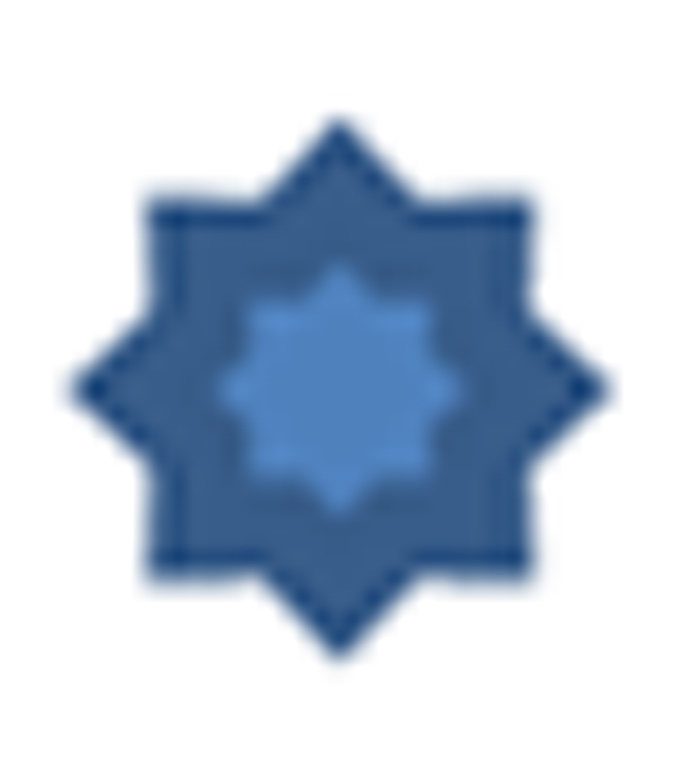
 UHRF1 or UHRF2 proteins.

To investigate if the increased level of DNMT3A proteins in the shUHRF2-expressing A549 cells is responsible for reduced tumor growth, we established A549 cell lines that stably expressed either the wild-type DNMT3A or its R882C mutant, which has an impaired enzymatic activity. The expression of DNMT3A or R882C mutant was confirmed by western blot analysis (Figure 7a) and the levels are comparable with the level of DNMT3A observed in shUHRF2-expressing A549 cells (Supplementary Figure S11C). HPLC analysis of total 5-meC revealed an approximately 40% (from ~3 to 4.2%) increase in DNA methylation in the DNMT3A but not in R882C mutant-expressing cells (Figure 7b). We further analyzed the global DNA methylation in DNMT3A expressing and control cells by reduced representative bisulfite sequencing (RRBS) assay. This analysis identified 2 725 891 CpG sites that were common in both samples and covered at least five times. As shown in Figure 7c, DNMT3A expression resulted in global increase of DNA methylation, generating substantially more hypermethylated (48 666) than hypomethylated (1 210) regions (Supplementary Figure S12A and B). To investigate how DNMT3A expression and its consequent change of DNA methylation affected gene expression, we also performed RNA-seq analysis for the control and DNMT3-expressing A549 cells (Supplementary Figure S12C). Although we did not detect a clear correlation of either differential promoter or gene body DNA methylation with gene expression (Supplementary Figure S12D and E), gene ontology analysis revealed enrichment for cell death and negative regulation of proliferation-related genes in 438 upregulated genes and enrichment for DNA replication-related genes in 307 downregulated genes in DNMT3-expressing cells (Figure 7d). To test how ectopic DNMT3A expression affected tumor growth, the control, DNMT3A and R882C mutant-expressing A549 cells were injected subcutaneously into nude mice and tumor growth was analyzed as above. As shown in Figure 7e and f, the tumors from the DNMT3A-expressing A549 cells were substantially smaller in both volume and weight than those from the control A549 cells, whereas the tumors from R882C mutant-expressing cells were similar to those from the control A549 cells. Immunohistochemistry staining confirmed the sustained expression of both wild type and mutant DNMT3A in the recovered tumors (Figure 7g). These data support the conclusion that increased DNMT3A expression and its associated increased DNA methylation are sufficient to impair tumor growth.

## Discussion

### UHRF1/2 promotes the fidelity of DNA methylation inheritance by negative regulation of *de novo* methylation

Given its central role in targeting DNMT1 to DNA replication forks [14, 15], UHRF1 has been well recognized as a protein essential for DNA maintenance methylation. This function is conserved in animals and plants, as the homolog of UHRF1 is required for DNA methylation in zebrafish and Arabidopsis [45]. Although the highly related UHRF2 is not required for DNA maintenance methylation [24, 25], in this study we provide compelling evidence that UHRF2 negatively regulates DNA methylation in variety of cell lines tested (Figures 1, 2 and 6 and Supplementary Figures S1, S2, 3 and S11). We demonstrated that UHRF2 negatively regulates DNA methylation by targeting DNMT3A for proteasome-dependent degradation (Figures 3, 4,5,6). To our surprise, much like UHRF2, UHRF1 also targets DNMT3A for proteasome-dependent degradation and negatively regulate DNA methylation by DNMT3A (Figures 3–6). Thus, both UHRF1 and UHRF2 negatively regulate *de novo* DNA methylation by targeting DNMT3A degradation in both normal somatic and cancer cells.

A recent study identified UHRF2 as a reader of 5-hydroxylmethyl C (5-hmC) [46], which is generated from 5-meC by TET family protein-catalyzed oxidation reaction. The ability of UHRF2 to bind 5-hmC and enhance TET1 enzymatic activity [46, 47] raises the possibility that UHRF2 may negatively regulate DNA methylation through TET1-mediated demethylation. However, we found that knockdown of Uhrf2 resulted in a comparable increased level of DNA methylation in the wild type and Tet1^−/−^ MEF cells (Supplementary Figure S13), which argues against the possibility that UHRF2 negatively regulates DNA methylation through TET1-mediated demethylation.

Our study raises an interesting question as to why in evolution the UHRF family proteins are endowed with the activities to simultaneously mediate DNA maintenance methylation and suppress *de novo* methylation by DNMT3A? DNA methylation patterns are thought to be set up in early mammalian embryonic development and then maintained faithfully during the division of somatic cells. The concept of maintenance and *de novo* methylation laid out in the original DNA methylation inheritance model has been well supported by experimental data. However, it has also become clear that the *de novo* enzymes DNMT3A and DNMT3B are not only essential for setting up DNA methylation patterns in early embryos, but also required for maintaining the specific DNA methylation patterns in somatic cells [11–13, 32]. In fact, a functional cooperation between maintenance methylation by DNMT1 and *de novo* methylation by DNMT3A/3B has been reported some years ago [10, 48, 49]. Thus, the revised model proposed that the maintenance of DNA methylation relies not only on the recognition of hemimethylated DNA by DNMT1 but also on the activities of DNMT3A/3B targeted to specific chromatin regions that contain methylated DNA [13]. However, the involvement of DNMT3A/3B in maintaining cellular DNA methylation patterns also raises the question as how the activities of DNMT1 and DNMT3A/3B are coordinated to favor the faithful inheritance of DNA methylation in mitosis, as excessive *de novo* methylation would result in increased DNA methylation and alteration of epigenetic inheritance. One mechanism for balancing the act of *de novo* and maintenance methylation is the control of their relative expression in different development stages, as DNMT3A and DNMT3B are highly expressed in early embryo but become downregulated in somatic cells, whereas the expression of DNMT1 is more or less constant over the same developmental period [7, 8]. Our study reveals a novel and unexpected mechanism that coordinates the activities of maintenance and *de novo* methylation. By endowing UHRF1, a protein essential for DNA maintenance methylation, and its highly related UHRF2 the ability to target DNMT3A for degradation, UHRF1/2 can suppress *de novo* methylation and thus enhance the fidelity of DNA methylation inheritance by favoring DNMT1-mediated maintenance methylation. Thus, we propose a new working model in which the UHRF family proteins control the fidelity of DNA methylation inheritance by balancing the activity of maintenance and *de novo* DNA methylation by DNMT3A (Figure 7h). According to this model, abrogation of this negative regulation on DNMT3A would lead to increased DNA methylation, as shown by knockdown of UHRF2 in NIH3T3, MEF and many other cell lines and by knockdown of Uhrf1 in Dnmt1^−/−^ ES cells in our study. However, this model also predicts that overexpression of UHRF1/2 would result in excessive degradation of DNMT3A and consequently DNA hypomethylation, as observed in cancers.

Our model is consistent with the idea that once the patterns of DNA methylation are established in early development, the priority for somatic cells would be to faithfully preserve methylation patterns by sustaining DNA maintenance methylation and suppressing *de novo* DNA methylation. Given that the UHRF family proteins are highly conserved in evolution, it will be interesting to determine whether the same mechanism operates in zebrafish in which only one UHRF1 homolog is identified. It is noteworthy that while both UHRF1 and UHRF2 have been shown to interact with DNMT3A and DNMT3B [24, 50], only DNMT3A is targeted for ubiqutination and degradation by UHRF1 and UHRF2. The mechanism for this differential effect on DNMT3A and DNMT3B also remains to be determined.

### UHRF1/2 overexpression as a mechanism for DNA hypomethylation in cancers and a driving force for tumorigenesis

DNA hypomethylation at a genome-wide scale or more prevalently in repetitive sequences is one of the most recognized and common epigenetic changes in cancer [27, 28, 51, 52]. Animal studies have shown that DNA hypomethylation as a consequence of Dnmt deficiencies promotes tumorigenesis in a tissue-dependent manner [29–31]. DNA hypomethylation is thus a causal factor and not a bystander of cancer. DNA hypomethylation may contribute to tumorigenesis through multiple mechanisms including changes in gene expression, loss of imprinting and increased genome instability [53–56]. However, since the initial seminal findings in 1983 [51, 52], the underlying mechanism for this widespread DNA hypomethylation in cancer remains largely unknown. Our finding that UHRF1/2 negatively regulates DNA methylation uncovers a mechanism for DNA hypomethylation in cancer. As discussed above, although negative regulation of *de novo* methylation by UHRF1/2 likely serves as a mechanism enhancing the fidelity of DNA methylation inheritance in somatic cells, excessive downregulation of DNMT3A by overexpressed UHRF1/2 would drive the gradual loss of DNA methylation and lead to DNA hypomethylation in cancer (Figure 7h). In this regard, UHRF1 overexpression is widespread in cancers (Figure 5c–e and Supplementary Figure S10), consistent with the previous reports that UHRF1 is overexpressed in various cancers including breast, prostate, lung, colorectal and bladder [17, 42, 44, 57–59]. It is noteworthy that the RNA-seq data indicate that both DNMT1 and DNMT3A are also overexpressed in most if not all cancers (Figure 5c–e and Supplementary Figure S10), consistent with previous observation for coordinated overexpression of DNMT3A, DNMT3B and DNMT1 in cancers. However, in contrast to overexpression of DNMT3A in transcripts, reduced DNMT3A proteins were observed in our study by western blot analysis of lung cancer specimens (Figure 5f). In addition, an inverse correlation between UHRF1/2 overexpression and DNMT3A proteins is also observed in lung cancer cell lines (Figure 6a). Furthermore, our studies with cancer cell lines demonstrated that UHRF1/2 actively downregulates DNMT3A in all cancer cell lines tested (Figures 3e, f and 6b). All these data are consistent with our working model in Figure 7h showing that the widespread UHRF1/2 overexpression leads to excessive degradation of DNMT3A proteins in cancers, which in turn leads to widespread hypomethylation in cancers. In this regard, it is not a coincidence that DNMT3A is shown to play a key role in maintaining methylation in repetitive sequences and DNA hypomethylation in cancer is generally observed in highly repetitive sequences.

It is noteworthy that during preparation of this manuscript, Mudbhary *et al*. [60] reported that UHRF1 overexpression in zebrafish hepatocytes drives DNA hypomethylation and hepatocellular carcinoma. They also showed that UHRF1 overexpression defines a subclass of aggressive human hepatocellular carcinoma characterized by genomic instability. They proposed that UHRF1 overexpression destabilizes and delocalizes DNMT1 and consequently leads to DNA hypomethylation. In our study we did not observe a significant effect of knocking down of UHRF1 and UHRF2 on the protein levels of DNMT1 (Figures 3a,e,f and 6b). Thus, the destabilization and mis-targeting reported in their study may operate under excessive UHRF1 overexpression, as they suggested, whereas the negative regulation of DNMT3A by UHRF1/2 reported in our study occurs in both regular and cancer cells.

Our study also supports a causal link between DNA hypomethylation and tumorigenesis. Although UHRF2 is less frequently overexpressed in cancers in comparison with UHRF1 (Figure 5c and d) and is therefore less responsible for downregulation of DNMT3A in cancers, in a few cancer cell lines, especially A549, UHRF2 appears to play a dominant role in negative regulation of DNMT3A. This allowed us to assess the extent of DNA hypomethylation and effect on tumor growth controlled by negative regulation of DNMT3A by UHRF1/2. Note that we could not directly evaluate the scope of DNA hypomethylation induced by overexpressed UHRF1 in cancer cells, because UHRF1 is also required for DNA maintenance methylation by DNMT1 and knockdown of UHRF1 compromises DNA methylation by DNMT1. Importantly, knockdown of UHRF2 in A549 cells led to increased global DNA methylation as well as methylation in LINE1, demonstrating that downregulation of DNMT3A by UHRF2 is causally linked to DNA hypomethylation phenotype in A549 cells. Furthermore, as knockdown of UHRF2 in A549 cells led to reduced cell proliferation and impaired tumor growth (Figure 6), our data support a positive link between DNA hypomethylation and tumor growth. In further support of this conclusion, we demonstrated that overexpression of wild type but not enzymatic activity-deficient DNMT3A mutant phenocopied the impaired tumor growth result observed for UHRF2 knockdown A549 cells (Figure 7e and f). Thus, knockdown of UHRF2 or overexpression of DNMT3A all led to increased global DNA methylation and methylation in repetitive sequences, reduced cell proliferation and reduced tumor growth, thus supporting that overexpression of UHRF1/2 is a mechanism driving DNA hypomethylation in cancer and that DNA hypomethylation promotes tumorigenesis.

Somatic recurrent DNMT3A mutations have been identified in multiple types of human hematological malignancies [33, 34], thus linking loss of DNMT3A enzymatic function directly with tumorigenesis. The widespread UHRF1/2 overexpression in cancers shown in this and previous studies provides an alternative and possibly more prevalent mechanism for inactivating or suppressing DNMT3A activity in cancers, which may in turn promote tumorigenesis through consequent DNA hypomethylation. Thus, UHRF1/2 represents a novel drug target for cancer therapy.

## Materials and Methods

### Plasmids and antibodies

Various UHRF1, UHRF2 and DNMT3A expression plasmids were constructed using either the pSG5-vector or pPYCAGIP vector for expression in cancer and ES cells. The oligos encoding shRNAs against human UHRF1 or UHRF2 or mouse Uhrf1 or Uhrf2 were cloned pSicoR-GFP or pSicoR-RFP vector. The pSicoR-RFP vector was generated by replacing the *GFP* gene with *RFP*. The shRNA sequences were as follows: shUhrf1A sense CCAGTTAACCAGGCATCTA, antisense TAGATGCCTGGTTAACTGG; shUhrf1B sense GATTGTTAACATATTGCAA, antisense TTGCAATATGTTAACAATC; shUhrf2A sense GGACTAATGGAAATGTAAA, antisense TTTACATTTCCATTAGTCC; shUhrf2B sense GAATGATGCTCAGGTTAAA, antisense TTTAACCTGAGCATCATTC; shUHRF1A sense AGGTGGTCATGCTCAACTA, antisense TAGTTGAGCATGACCACCT; shUHRF2B sense GGTGGAATTCATGGTCGAA, antisense TTCGACCATGAATTCCACC. All plasmids were verified by DNA sequencing. The antibodies used included UHRF1(AbMART, Shanghai, China), UHRF2 (homemade), H3K9me3 (Abcam, Cambridge, MA, USA), DNMT1(AbMART), DNMT3A, DNMT3B, Flag (Sigma-Aldrich, Shanghai, China), Flag (HuaAn Corporation, Hangzhou, China) and methyl-C (Eurogentec, Seraing, Belgium).

### Cell culture, transient transfection and immunofluorescence staining

The *Uhrf1*^−/−^, *Dnmt1*^−/−^ and *Dnmt3ab*^−/−^ ES cells were routinely cultured with Dulbecco's modified Eagle's medium (plus 15% fetal bovine serum (Biochrom, Berlin, Germany)) medium on feeder cells. NIH3T3, HeLa and 293T cells were routinely maintained with regular Dulbecco's modified Eagle's medium plus 10% fetal bovine serum (Gibco, Waltham, MA, USA). Transient transfections were carried out using Lipofectamine 2000 (Invitrogen, Waltham, MA, USA) essentially according to the manufacturer’s instruction.

Immunofluorescence staining for various proteins was carried out essentially as described [20].

### Analysis of DNA methylation by immunofluorescent staining, HPLC and bisulfite sequencing

The analysis of DNA methylation by immunostaining with 5-meC antibody, HPLC and bisulfite sequencing is as described [20]. The quantification of the percentage of cells with change of DNA methylation was based on counting of more than 200 cells for each experiment. We compared the DNA methylation intensity by visual inspection between cells transfected with shRNAs and adjacent untransfected control cells. For the shUHRF1-transfected cells, the percentage represents the cells with reduced DNA methylation, whereas for the shUHRF2-transfected cells it represents the cells with increased DNA methylation.

### *In vitro* and *in vivo* ubiquitination assays

For *in vitro* ubiquitination assays, GST-UHRF1 and GST-UHRF2 and their mutants were expressed and purified from *Escherichia coli* and 6xHis-DNMT3A and DNMT3A2 were expressed and purified from insect cells. The E1 proteins were kindly provided by Dr Yanhui Xu (Fudan University). E2 (UBC5c) was expressed and purified from bacteria and Ub purchased from BostonBiochem (Cambridge, MA, USA). A typical reaction mixture contained 200 ng E1, 400 ng E2, 10 ug GST-UB, 600 ng UHRF1 or UHRF2 and 800 ng DNMT3A in ubiquitination reaction buffer (50 mmTris-Cl (pH 7.5), 5 mm MgCl_2_, 1 mm dithiothreitol (DTT) and 4 mm ATP). The mixture was incubated at 37 °C for 1 h, and the reaction was stopped by adding an equal volume of 2× sodium dodecyl sulfate loading buffer. The reaction was resolved by sodium dodecyl sulfate polyacrylamide gel electrophoresis, followed by western blotting.

For detecting endogenous DNMT3A ubiquitination in 293T cells, UHRF1 or UHRF2 and their mutants were co-transfected with 6xHis-Ub into 293T cells. Two days after transfection, the cells were treated with 20 μm MG132 for 8 h and then harvested for preparation of whole-cell extracts. Ubiquitinated DNMT3A was detected by western blot using anti-DNMT3A antibody.

### Analysis of pairwise RNA-seq data from the TCGA database

RNA-seq data were downloaded for nine cancers from The Cancer Genome Atlas (TCGA) database (TCGA public data until 17 July 2015). The gene expression was analyzed by RSEM data in pairs of matched benign and malignant tissue (from the same patient) using a paired Wilcoxon signed-rank test as described [61].

### RRBS and data analysis

RRBS assay for genomic DNA prepared from the DNMT3A expressing and control A549 cell lines were essentially as described [62]. RRBS reads were mapped to human genome (hg19) by using Bismark (v0.12.20) [63]. Only CpGs with at least five reads coverage were used for subsequent analyses. The CpG methylation level was represented by C/C+T. R package methylKit [64] was used to find differential methylated regions. Here we used 1 kb tilling region across whole genome to find differential methylated regions to increase statistical power.

### RNA-seq and data analysis

RNA-seq analysis for RNA samples prepared from the DNMT3A expressing and control A549 cell lines were performed by BerryGenomics (Beijing, China). RNA-seq reads were mapped to human genome (hg19) by using TopHat (v1.4.1) [65] and gene expression levels were calculated by Cufflinks (v2.2.1) [66]. Only genes with FPKM>1 were kept. Differentially expressed genes were identified by Cuffdiff (v2.2.1) [67] with false discovery rate (FDR)<0.05.

### Analysis of lung cancer specimens

The study with lung cancer samples was approved by the local ethic committees in Shanghai Cancer Hospital, Fudan University and all the clinical specimens including lung cancer samples and paired normal lung tissues were collected with the written consent from patients.

### The analysis of growth of xenograft tumors in nude mice

The groups of 4-week-old male nude mice (*n*=7) were each injected subcutaneously with 5 million cells of control A549 or A549 cells expressing either shUHRF2 or the wild type or R882C mutant DNMT3A as indicated. A few mice died during the experiment and were removed. After 6 weeks, the mice were killed and tumors were extirpated, weighed and photographed.
