# Supplementary material for: Negative regulation of DNMT3A de novo DNA methylation by frequently overexpressed UHRF family proteins as a mechanism for widespread DNA hypomethylation in cancer
Source: Cell Discov. 2016 Apr 12;2:16007–. doi: 10.1038/celldisc.2016.7 (PMC4849474; doi:10.1038/celldisc.2016.7)
Supplement: Supplementary Figure S1 [file celldisc20167-s1.pdf]

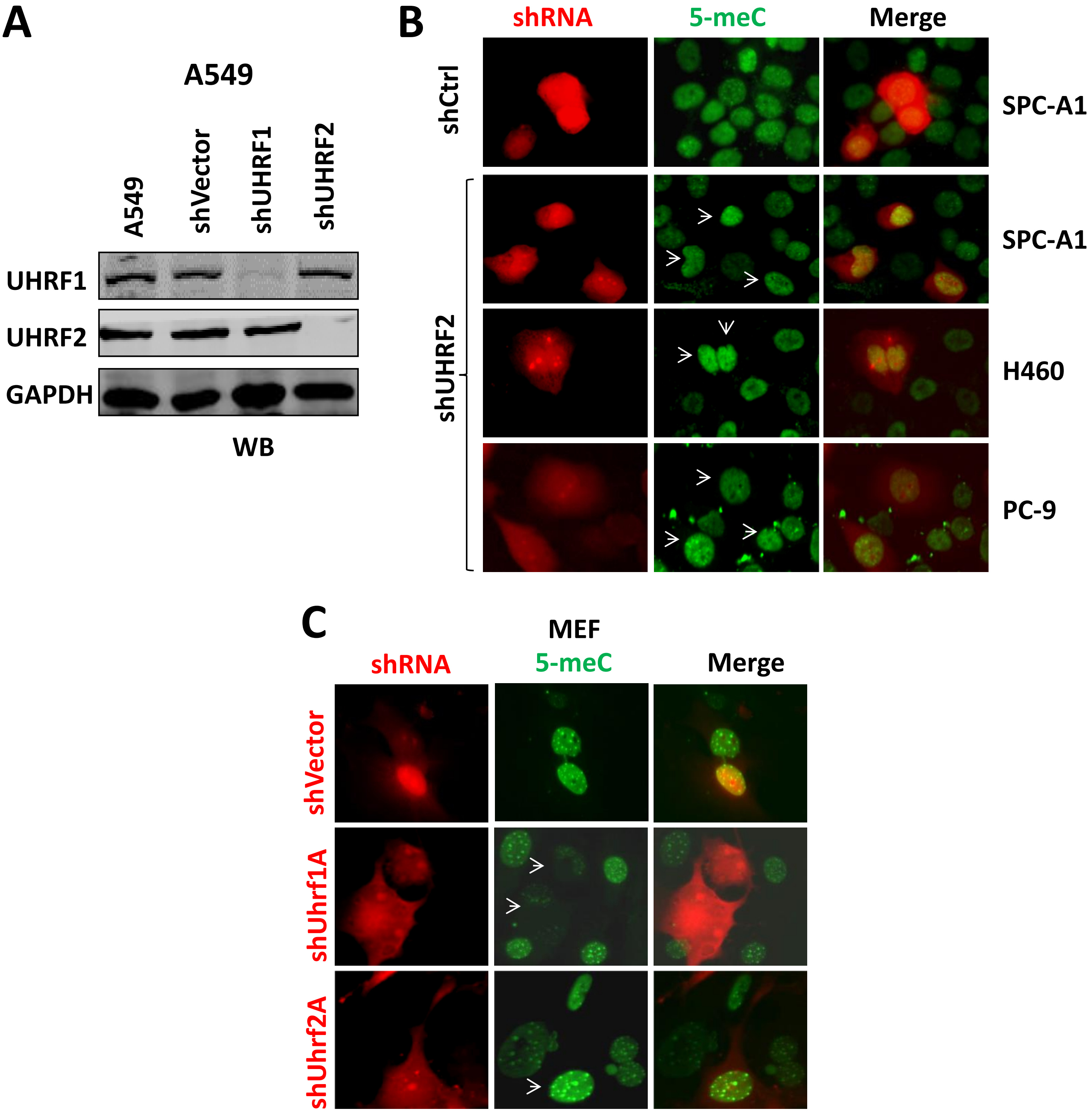

Supplementary Figure. S1. Knockdown of UHRF2 results in increased levels of DNA methylation in various cell lines. (A) A549 cells were infected with lentiviruses encoding the control or shUHRF1 or shUHRF2 as indicated and the whole cell extracts were prepared and analyzed by Western blot using antibodies as indicated. (B-C)) Different human or mouse cell lines were transfected with shRNAs as indicated and the levels of DNA methylation were examined by immunostaining using anti-5meC antibody. Note that increased 5-meC was observed in SPC-A1, H460 and PC-9 cells transfected with shUHRF2 (red) (B) or the primary mouse MEF cells transfected with shUhrf2 (C).
