# Supplementary material for: Negative regulation of DNMT3A de novo DNA methylation by frequently overexpressed UHRF family proteins as a mechanism for widespread DNA hypomethylation in cancer
Source: Cell Discov. 2016 Apr 12;2:16007–. doi: 10.1038/celldisc.2016.7 (PMC4849474; doi:10.1038/celldisc.2016.7)
Supplement: Supplementary Figure S3 [file celldisc20167-s3.pdf]

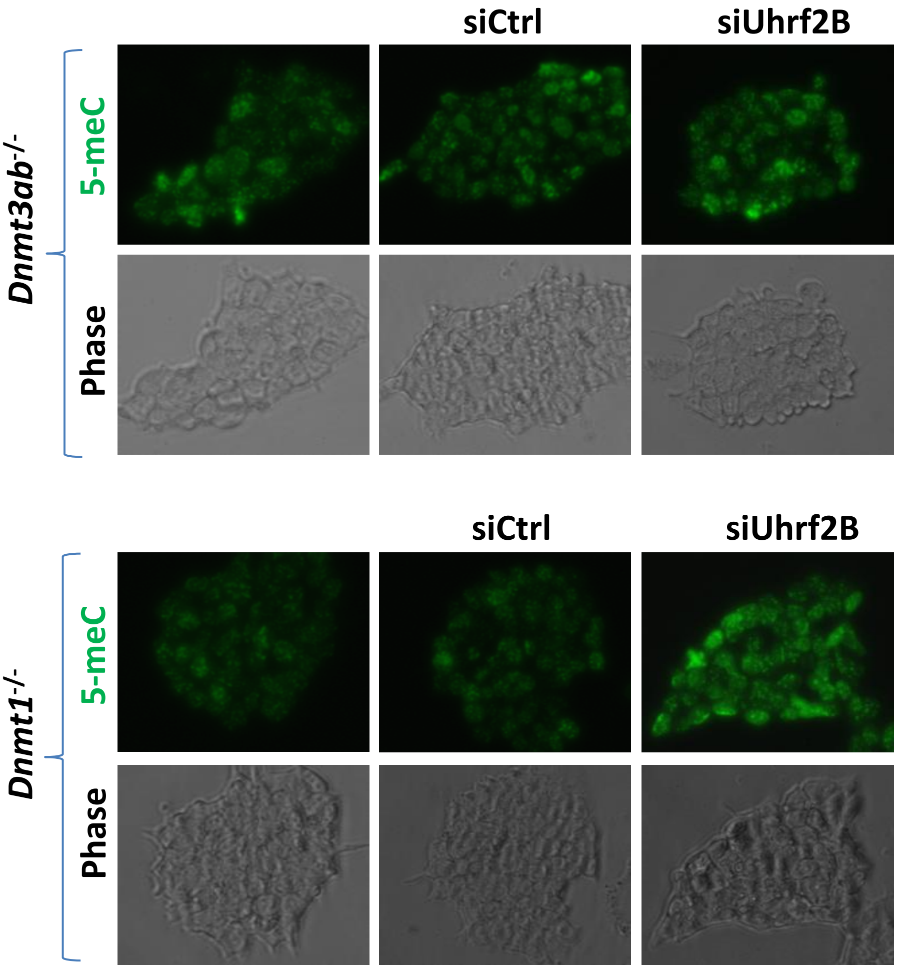

Supplementary Figure S3. Knockdown of Uhrf2 by siRNA results in elevated levels of 5-meC in the *Dnmt1-/-* but not *Dnmt3ab-/-* ES cells. The *Dnmt*1-/- and *Dnmt3ab-/-* ES cells were transfected with control siRNA (siCtrl) or siUhrf2B and three days after transfection the cells were proceeded for immunostaining analysis of 5-meC. All images were acquired with an Olympus TH4-20D microscope system under the exact same condition
