# Supplementary material for: Negative regulation of DNMT3A de novo DNA methylation by frequently overexpressed UHRF family proteins as a mechanism for widespread DNA hypomethylation in cancer
Source: Cell Discov. 2016 Apr 12;2:16007–. doi: 10.1038/celldisc.2016.7 (PMC4849474; doi:10.1038/celldisc.2016.7)
Supplement: Supplementary Figure S4 [file celldisc20167-s4.pdf]

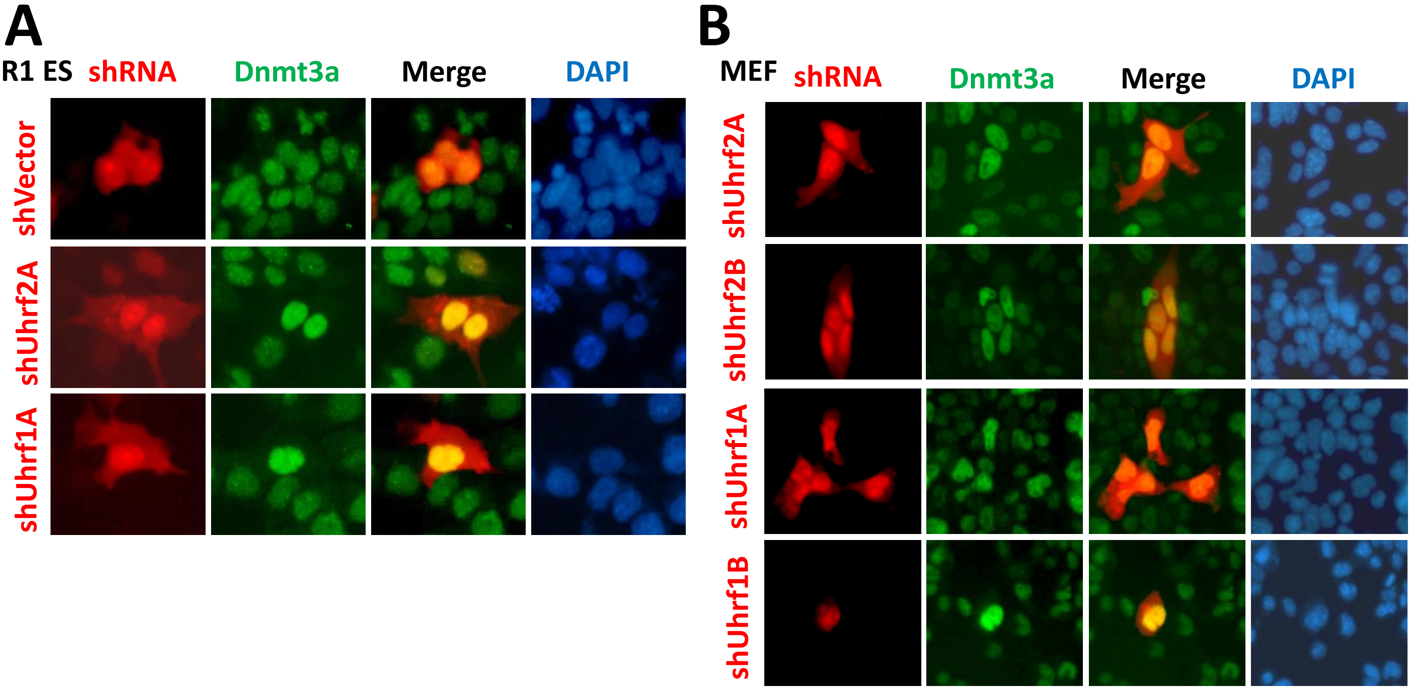

Supplementary Figure S4. Knockdown of Uhrf1 and Uhrf2 in R1 ES cells or MEF cells results in elevated levels of Dnmt3a proteins. (A) The mouse R1 ES cells were transfected with indicated shRNA constructs and analyzed for the effect on Dnmt3a by immunostaining. Note that increased levels of Dnmt3a were observed in cells transfected with either shUhrf2 or shUhrf1, but not the control vector. Also shown are merged figure and DAPI staining. (B) The mouse MEF cells were transfected with indicated shRNA constructs and analyzed for the effect on Dnmt3a by immunostaining as above. Again increased levels of Dnmt3a were observed in cells transfected with either shUhrf2 or shUhrf1.
