# Supplementary material for: Negative regulation of DNMT3A de novo DNA methylation by frequently overexpressed UHRF family proteins as a mechanism for widespread DNA hypomethylation in cancer
Source: Cell Discov. 2016 Apr 12;2:16007–. doi: 10.1038/celldisc.2016.7 (PMC4849474; doi:10.1038/celldisc.2016.7)
Supplement: Supplementary Figure S5 [file celldisc20167-s5.pdf]

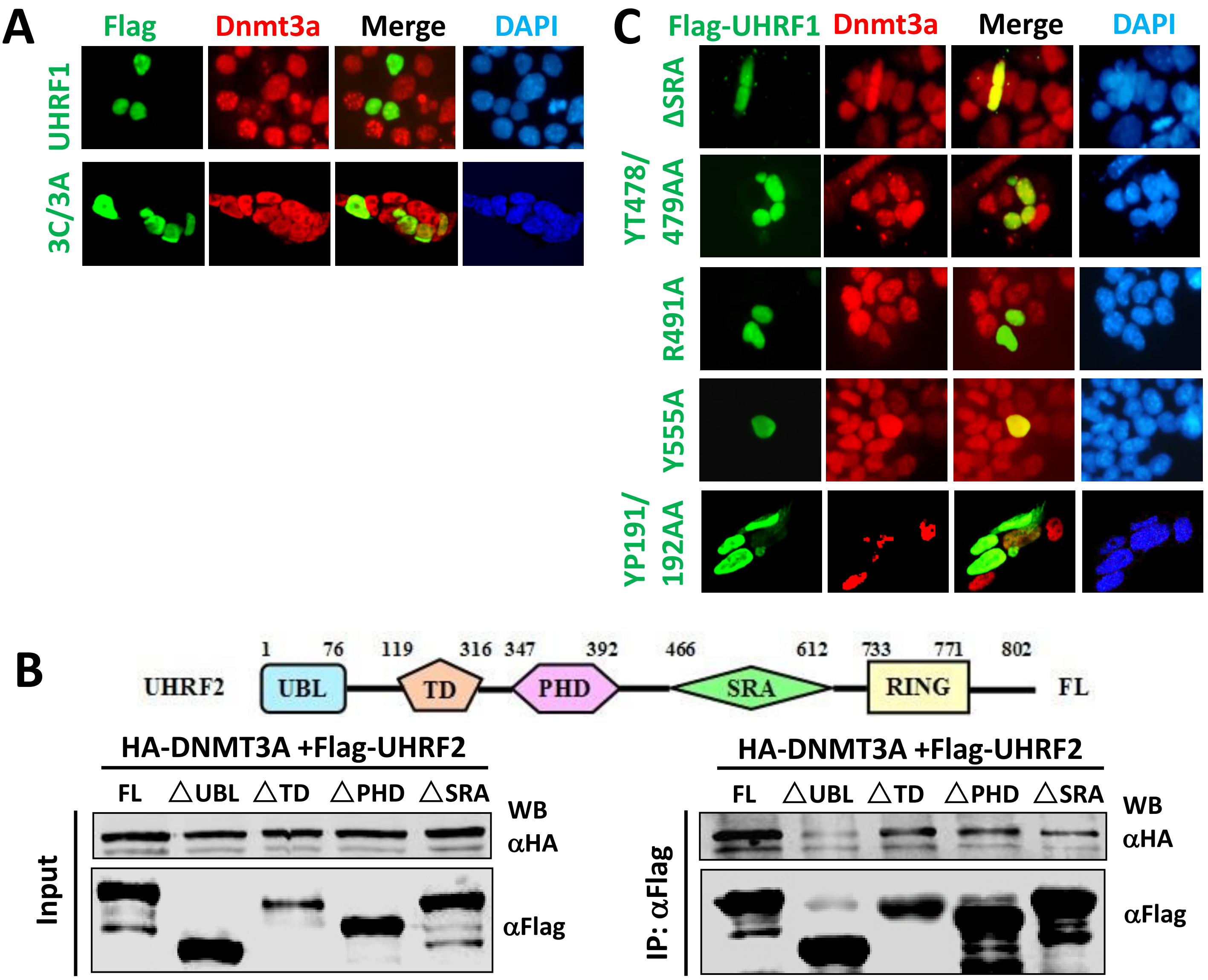

Supplementary Figure S5. The SRA domain is not required for the interaction between UHRF1/2 and DNMT3A but required for UHRF1/2-induced DNMT3A degradation. (A) The RING E3 ligase activity is required for UHRF1-induced Dnmt3a degradation in R1 ES cells. The R1 ES cells were transfected with Flag-tagged UHRF1 wild-type or 3C/3A mutant defective in RING E3 ligase activity and subsequently analyzed for Dnmt3a proteins by immunostaining. (B) IP-Western analysis showing that the UBL domain of UHRF2 is required for the interaction between UHRF2 and DNMT3A. The Flag-tagged full-length and various UHRF2 deletion mutants were co-expressed with HA-tagged DNMT3A in 293T cells by transient transfection. The IP-Western was performed using antibodies as indicated. (C) Mutations in SRA but not TUDOR domain impaired UHRF1-induced DNMT3A degradation in mouse E14 ES cells. E14 cells were transfected with Flag-tagged UHRF1 or mutants and IF was performed with anti-Flag and DNMT3A antibodies as indicated. The YP191/192AA mutation in the TUDOR domain is impaired in binding H3K9me2/3 and YT478/479AA, R491A and Y555A mutations in the SRA were impaired in binding hemi-mCpG.
