# Supplementary material for: Negative regulation of DNMT3A de novo DNA methylation by frequently overexpressed UHRF family proteins as a mechanism for widespread DNA hypomethylation in cancer
Source: Cell Discov. 2016 Apr 12;2:16007–. doi: 10.1038/celldisc.2016.7 (PMC4849474; doi:10.1038/celldisc.2016.7)
Supplement: Supplementary Figure S6 [file celldisc20167-s6.pdf]

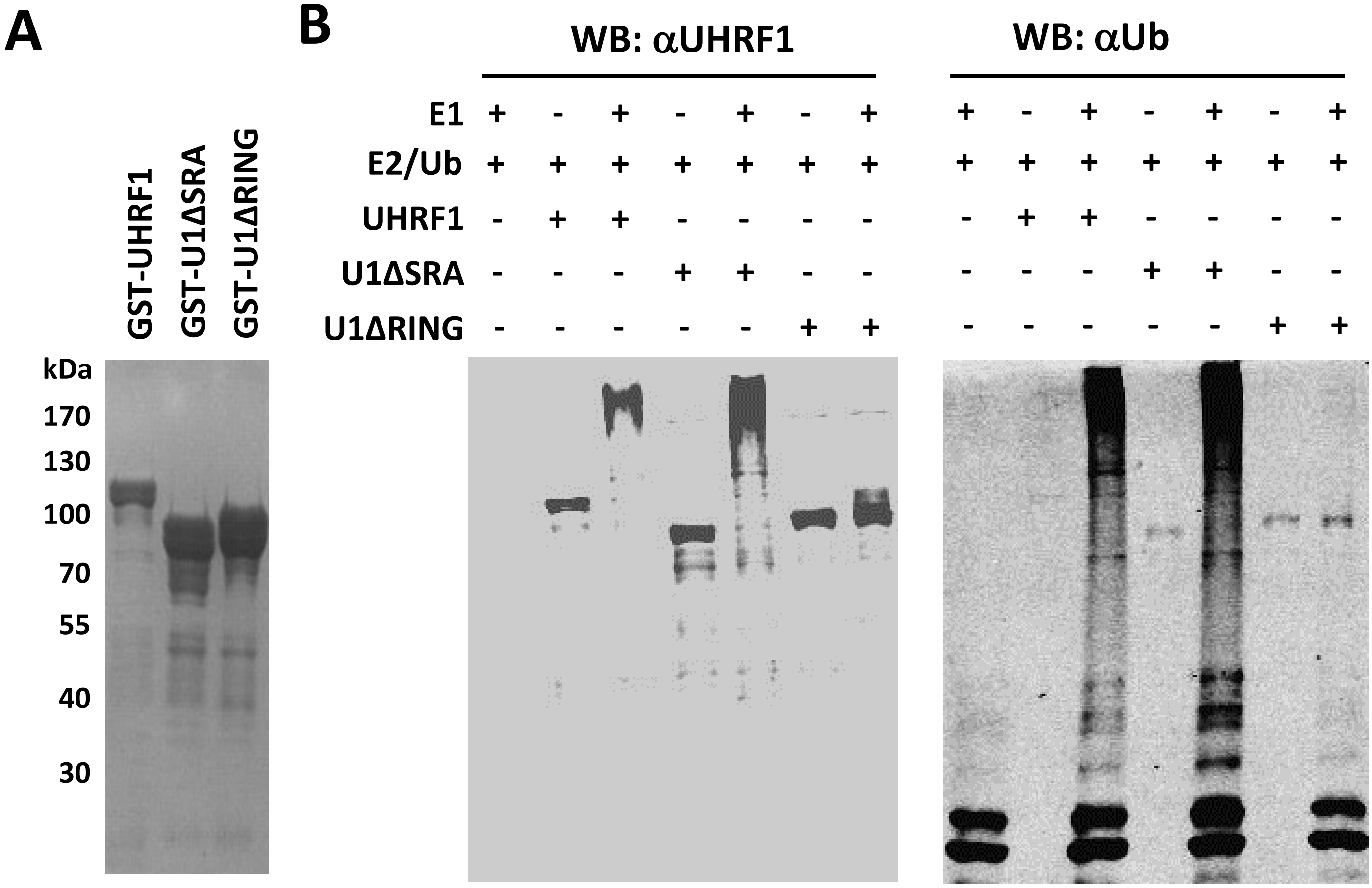

Supplementary Figure S6. The UHRF1 ubiquitin E3 ligase activity depends on its RING Finger domain but not the SRA domain. (A) Coomassie blue-staining showing purified GST- fusion proteins of the full-length UHRF1, deletion of SRA domain (ΔSRA) and deletion of RING (ΔRING). All three proteins were expressed and purified from E.coli and used in subsequent ubiquitination assays. (B) In vitro ubiquitin reactions examining the auto-ubiquitination activities of the purified wild-type and deletion mutants of UHRF1. The ubiquitin reactions were set up as indicated with Ubc5a as E2 and the ubiquitination of UHRF1 and mutants was revealed by Western blot analysis using an anti-UHRF1 antibody (left panel). The same reactions were also analyzed by Western blot using an anti-ubiquitin antibody (right panel). The results showed that both the wild-type and ΔSRA mutant exhibited an auto-ubiquitination activity, whereas the ΔRING mutant did not.
