# Supplementary material for: Negative regulation of DNMT3A de novo DNA methylation by frequently overexpressed UHRF family proteins as a mechanism for widespread DNA hypomethylation in cancer
Source: Cell Discov. 2016 Apr 12;2:16007–. doi: 10.1038/celldisc.2016.7 (PMC4849474; doi:10.1038/celldisc.2016.7)
Supplement: Supplementary Figure S7 [file celldisc20167-s7.pdf]

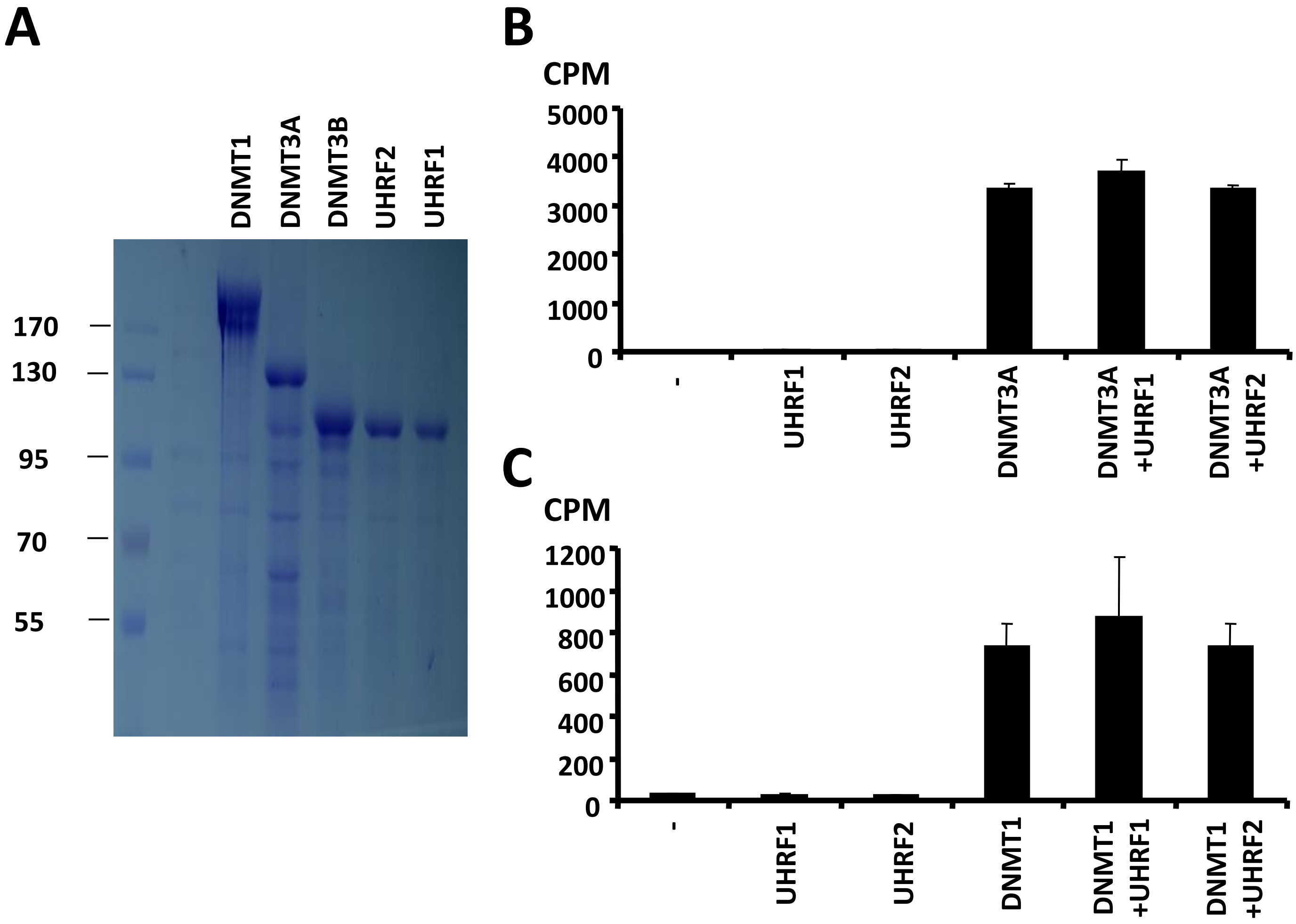

Supplementary Figure S7. UHRF1 and UHRF2 do not appear to significantly affect DNMT3A enzymatic activity *in vitro*. (A) Coomassie blue staining gel showing affinity-purified proteins used for in *vitro* assay. All proteins were expressed as 6x His tagged fusions and purified from F9 insect cells using Ni-NTA His-Bind resin according to manufacturer's instruction. (B) *In vitro* methylation reactions were performed with biotinylated oligonucleotide substrates and [methyl-^{3}H]-AdoMet as methyl donor according to Roth and Jeltsch 2000. The amount of proteins used in the reactions: 0.5 μg UHRF1, 0.5 μg UHRF2, and 0.5 μg DNMT3A. (C) The *in vitro* methylation assays were performed as above except DNMT1 was used. The amount of proteins used in the reactions: 0.5 μg UHRF1, 0.5 μg UHRF2, and 0.5 μg DNMT1.
