# Supplementary material for: Negative regulation of DNMT3A de novo DNA methylation by frequently overexpressed UHRF family proteins as a mechanism for widespread DNA hypomethylation in cancer
Source: Cell Discov. 2016 Apr 12;2:16007–. doi: 10.1038/celldisc.2016.7 (PMC4849474; doi:10.1038/celldisc.2016.7)
Supplement: Supplementary Figure S8 [file celldisc20167-s8.pdf]

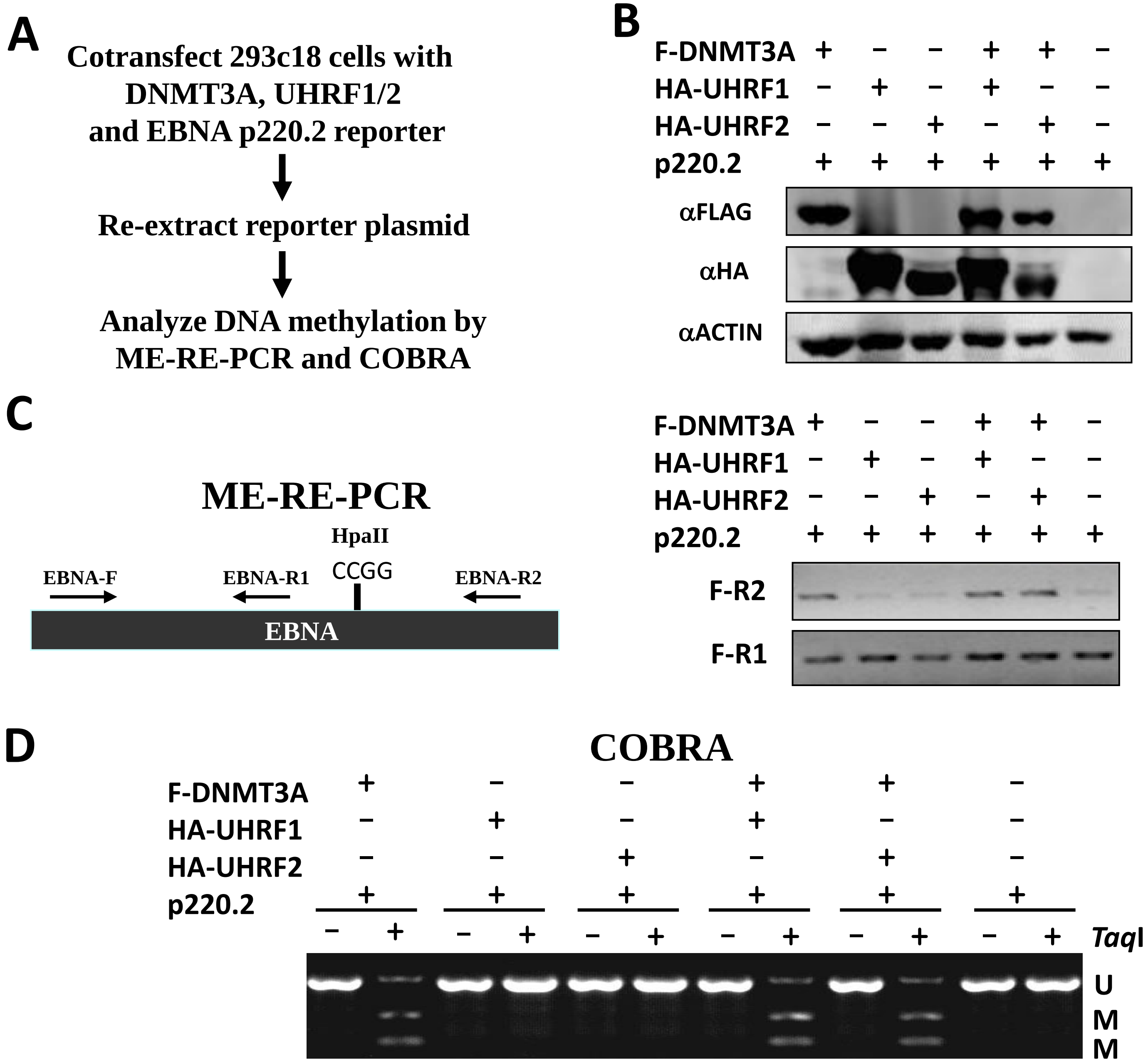

Supplementary Figure S8. UHRF1 and UHRF2 do not appear to significantly affect DNMT3A enzymatic activity *in vivo*. (A) Schematic illustration for the assay used for analyzing the effect of UHRF1 and UHRf2 on DNMT3A de novo DNA methylation activity in 293c18 cells. The assays were performed essentially as described by Hsieh 1999 (see references). (B) The expression of transfected constructs was verified by western blot analysis. (C) Analyzing the effect of co-expressed UHRF1 or UHRF2 on de novo DNA methylation of p220.2 reporter plasmid by DNMT3A by ME-RE-PCR. The methylation of the CG dinucleotide within the HpaII recognition site CCGG by DNMT3A would protect the reporter DNA from HpaII digestion. The F-R2 PCR product represented de novo methylation of the CCGG site by DNMT3A. Note co-expression of UHRF1 or UHRF2 with DNMT3A did not significantly affect the amount of F-R2 PCR product. The F-R1 PCR served as an internal control. (D) COBRA assay confirmed insignificant effect of co-expressed UHRF1 or UHRF2 on DNMT3A de novo DNA methylation activity.
