# Supplementary material for: Negative regulation of DNMT3A de novo DNA methylation by frequently overexpressed UHRF family proteins as a mechanism for widespread DNA hypomethylation in cancer
Source: Cell Discov. 2016 Apr 12;2:16007–. doi: 10.1038/celldisc.2016.7 (PMC4849474; doi:10.1038/celldisc.2016.7)
Supplement: Supplementary Figure S9 [file celldisc20167-s9.pdf]

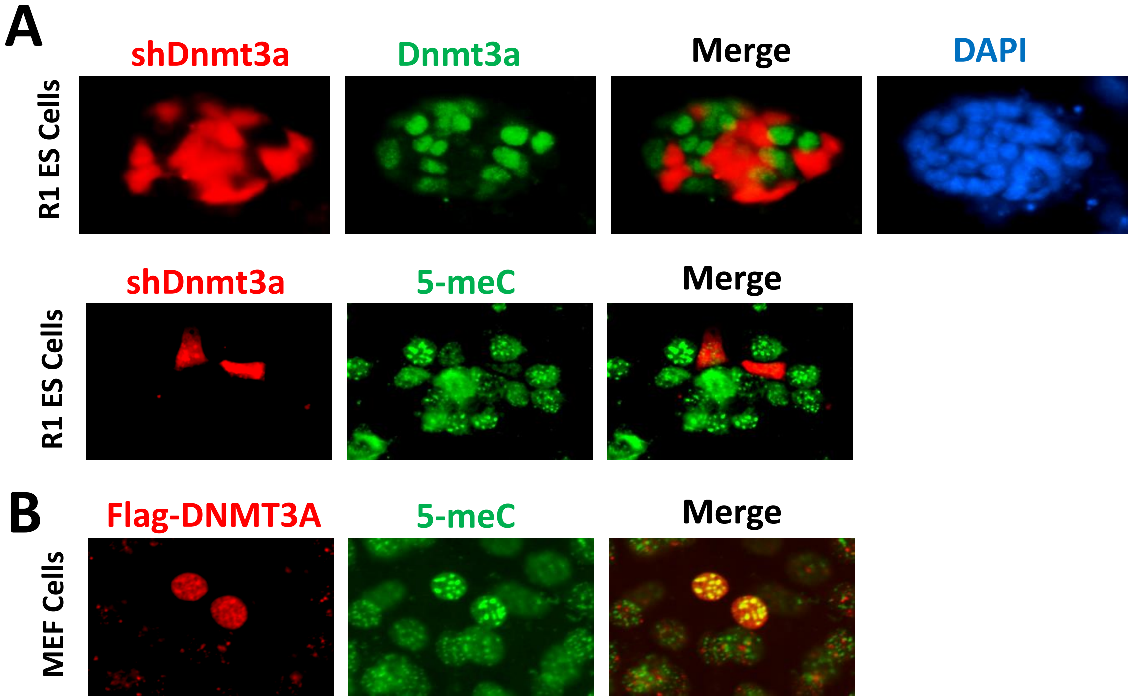

Supplementary Figure S9. Altering the levels of Dnmt3a in mouse R1 ES or MEF cells leads to global change of DNA methylation. (A) Knockdown of Dnmt3a in R1 ES cells led to diminished DNA methylation. The R1 ES cells were transfected with shDnmt3a plasmid and three days after transfection the cells were processed for immunostaining for Dnmt3a or 5-meC as indicated. Note that the shDnmt3a-transfected cells exhibited efficient down-regulation of Dnmt3a (top panel) and substantially reduced levels of 5-meC staining (low panel). (B) Ectopic expression of Flag-DNMT3A in MEF cells resulted in increased levels of DNA methylation. The primary MEF cells were transfected with plasmid encoding Flag-DNMT3A and three days after transfection the cells were processed for double immunostaining for 5-meC and Flag-DNMT3A. Note that the Flag-DNMT3A-positive cells showed enhanced levels of 5-meC staining.
