# Supplementary material for: Negative regulation of DNMT3A de novo DNA methylation by frequently overexpressed UHRF family proteins as a mechanism for widespread DNA hypomethylation in cancer
Source: Cell Discov. 2016 Apr 12;2:16007–. doi: 10.1038/celldisc.2016.7 (PMC4849474; doi:10.1038/celldisc.2016.7)
Supplement: Supplementary Figure S10 [file celldisc20167-s10.pdf]

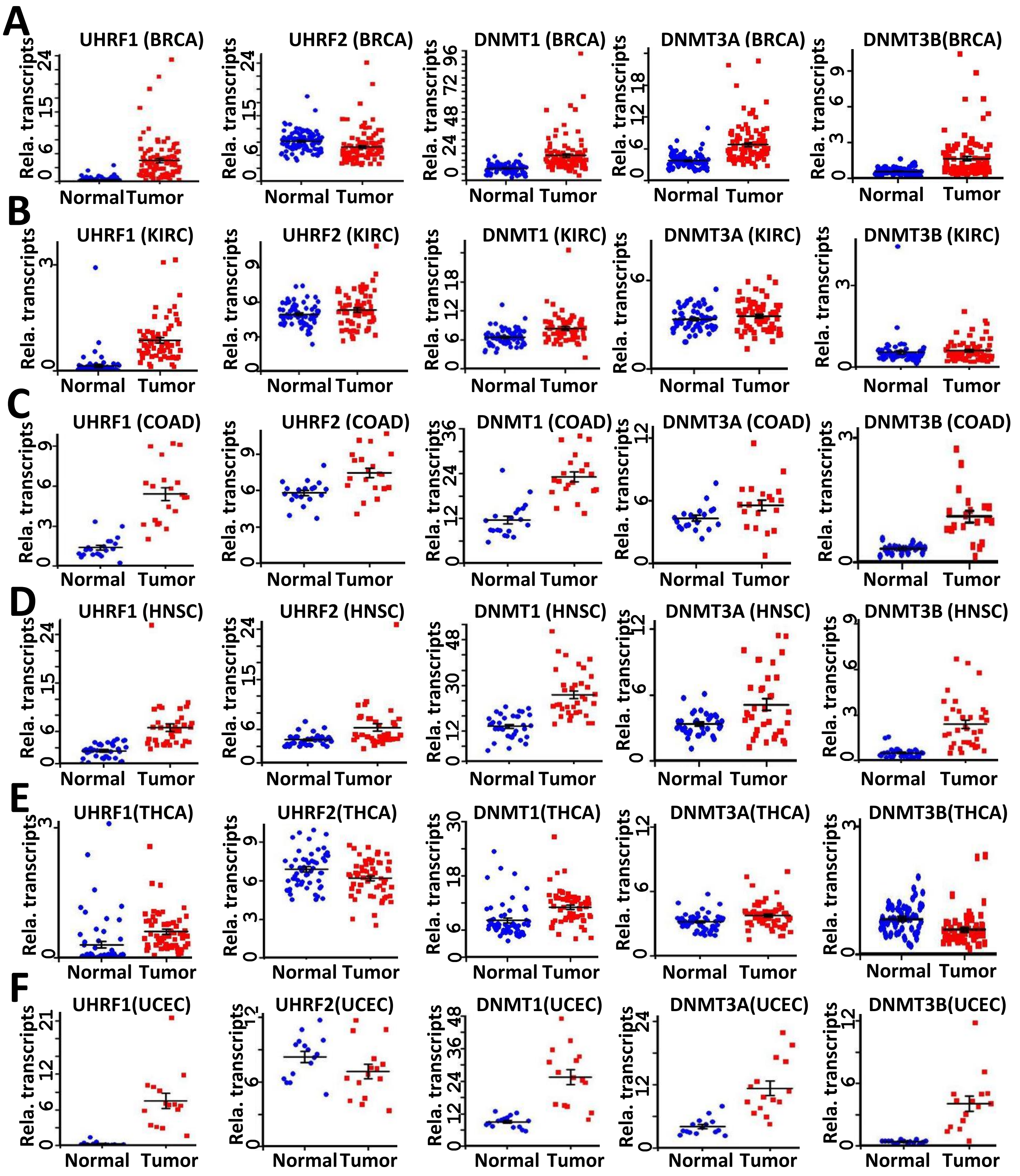

Supplementary Fig. S10. The relative levels of UHRF1, UHRF2, DNMT1, DNMT3A and DNMT3B transcripts in normal controls and tumors in various types of cancers. The RNA-seq data are from the TCGA database. (A) Breast cancer (BRCA); (B) Clear cell carcinoma (KIRC); (C) Colon and rectal adenocarcinoma (COAD); (D) Head and neck squamous cell carcinoma(HNSC); (E) Thyroid carcinoma(THCA); (F) Uterine corpus endometrial carcinoma(UCEC).
