# Supplementary material for: Negative regulation of DNMT3A de novo DNA methylation by frequently overexpressed UHRF family proteins as a mechanism for widespread DNA hypomethylation in cancer
Source: Cell Discov. 2016 Apr 12;2:16007–. doi: 10.1038/celldisc.2016.7 (PMC4849474; doi:10.1038/celldisc.2016.7)
Supplement: Supplementary Figure S11 [file celldisc20167-s11.pdf]

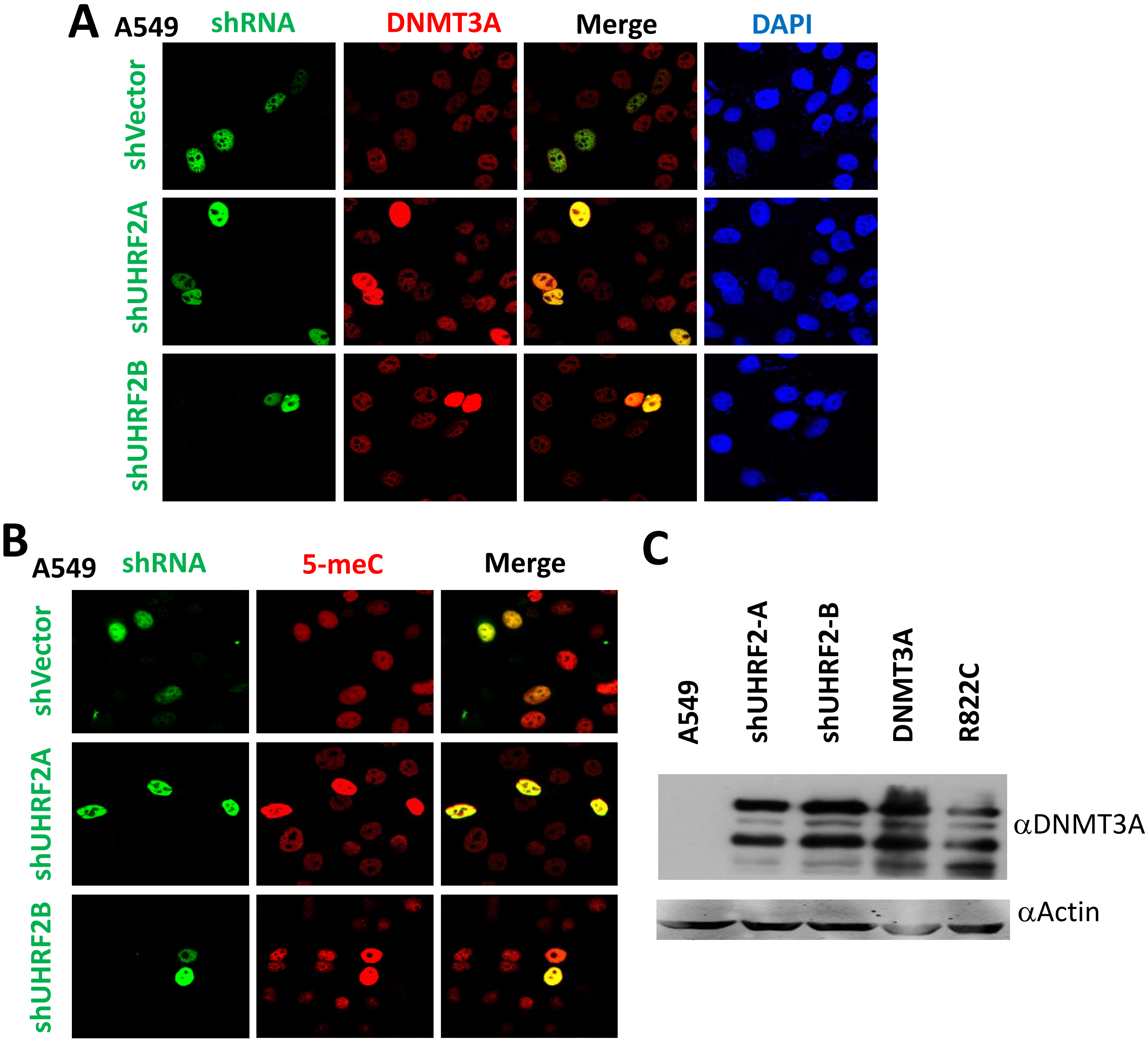

Supplementary Figure S11. Knockdown of UHRF2 in A549 cells results in increased levels of DNMT3A and elevated levels of DNA methylation. (A) A549 cells were transfected with control shRNA vector and two different shUHRF2 and three days after transfection the cells were processed for immunostaining analysis of DNMT3A. (B) The A549 cells were transfected as above and immunostaining analysis was performed for 5-meC. (C) Comparison of the levels of DNMT3A in A549 cells stably expressing two different shUHRF2 with those in A549 cells ecotopically expressing the wild-type or R822C DNMT3A mutant. Note the levels of DNMT3A are comparable among the four cell lines. Actin serves as a loading control.
