# Supplementary material for: Negative regulation of DNMT3A de novo DNA methylation by frequently overexpressed UHRF family proteins as a mechanism for widespread DNA hypomethylation in cancer
Source: Cell Discov. 2016 Apr 12;2:16007–. doi: 10.1038/celldisc.2016.7 (PMC4849474; doi:10.1038/celldisc.2016.7)
Supplement: Supplementary Figure S12 [file celldisc20167-s12.pdf]

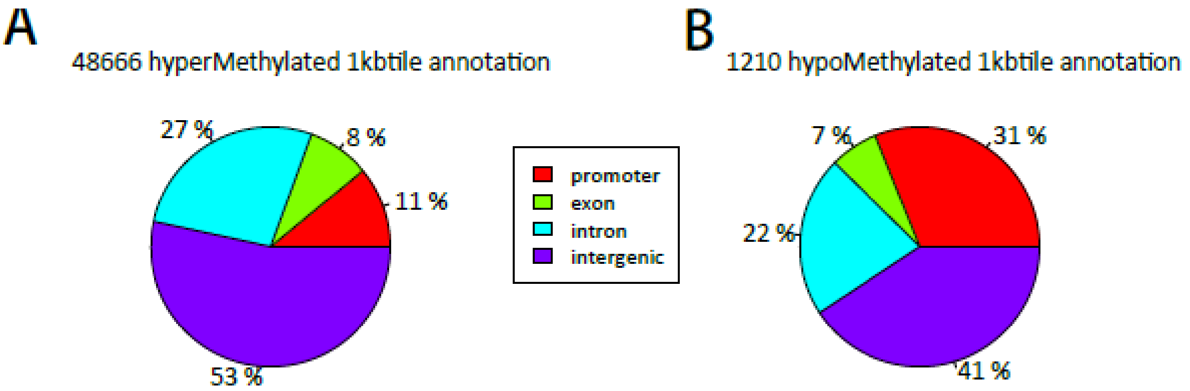

C

RNA Seq data summary

Sample	Total Reads	Unique Mapped Reads	Mapping Ratio
DNMT3A	23,789,848	17,724,841	74.5%
vector	26,241,416	19,131,455	72.9%

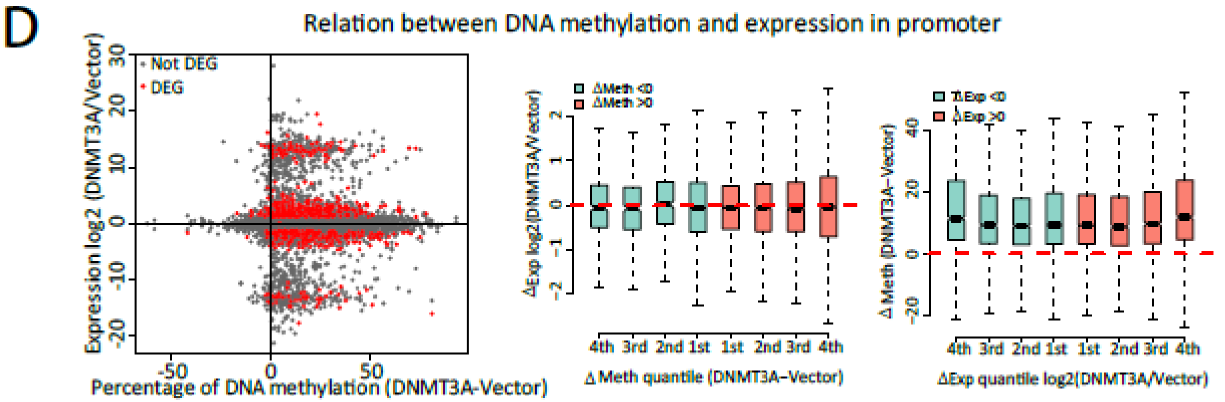

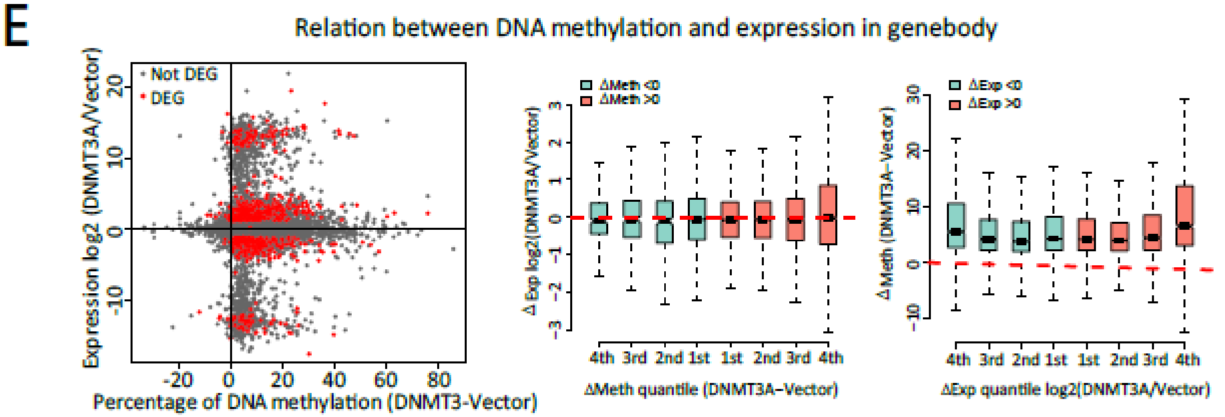

Supplementary Figure S12. Lack of clear correlation between DNMT3A-induced DNA methylation and gene expression. (A) Genomic annotation of hyper and (B) hypomethylated regions in DNMT3A vs. vector control A549 cells. 1kb tilling region was used to find DMRs (FDR<0.05). (C) Summary statistics for the RNA-seq data. (D) Relationship between promoter methylation and gene expression and (E) Relationship between genebody methylation and gene expression. In the scatter plot, x axis is methylation difference in promoter or genebody regions (DNMT3A minus vector), y axis is expression difference for the associated genes (log2(DNMT3A FPKM)-log2 (Vector FPKM)). In the left box plot, genes are separated into 2 groups: hypermethylated and hypomethylation. For each group, genes were divided into four quantile groups. Each box represents gene expression difference in each quantile. The right panel is similarly plotted except methylation and expression are reversed.
