# Supplementary material for: Negative regulation of DNMT3A de novo DNA methylation by frequently overexpressed UHRF family proteins as a mechanism for widespread DNA hypomethylation in cancer
Source: Cell Discov. 2016 Apr 12;2:16007–. doi: 10.1038/celldisc.2016.7 (PMC4849474; doi:10.1038/celldisc.2016.7)
Supplement: Supplementary Figure S13 [file celldisc20167-s13.pdf]

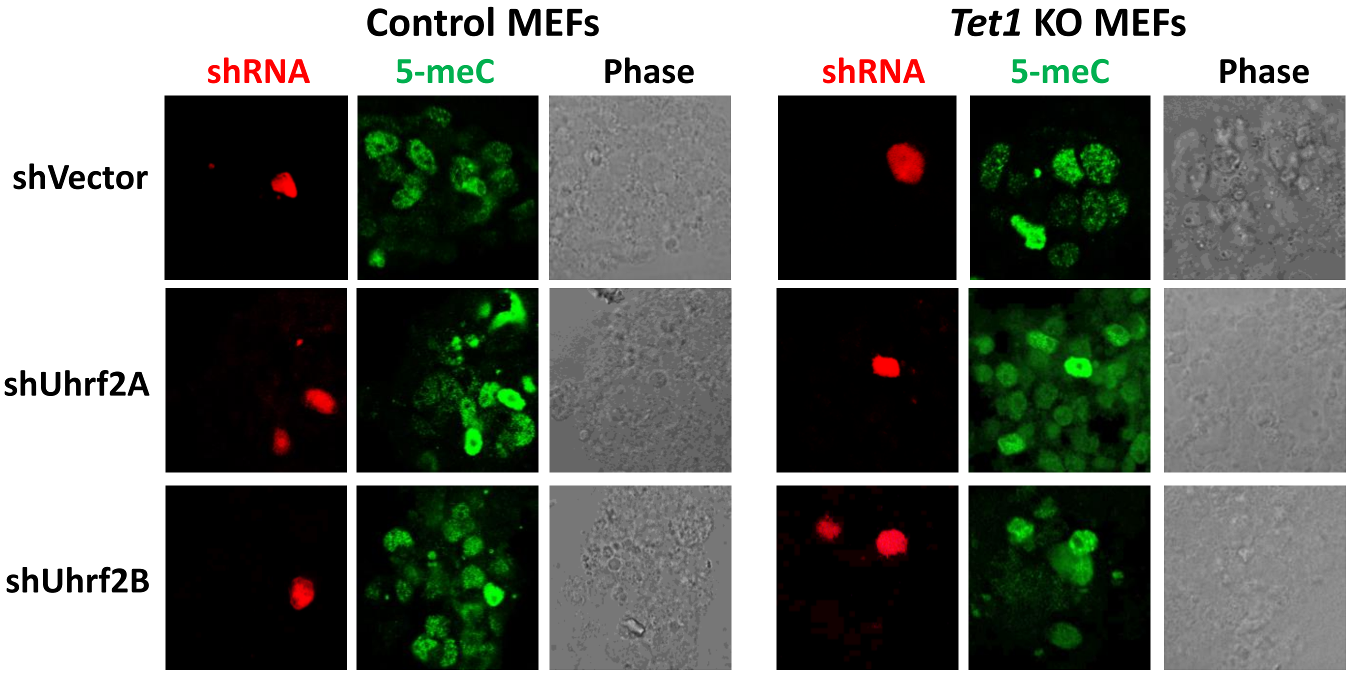

Supplementary Figure S13. Increased DNA methylation resulting from Uhrf2 knockdown in MEF cells is independent of Tet1. The control and *Tet1-/-* mouse fibroblast cells were transfected with control or shRNAs (red) against Uhrf2 as indicated. Three days after transfection the cells were processed for immunofluorecent staining for 5-meC (green). Also shown were the phase contrast images of the cells. Note that knockdown of Uhrf2 in both wild-type and *Tet1-/-* MEFs led to increased DNA methylation, indicating that the increased DNA methylation resulting from Uhrf2 knockdown is unlikely due to its potential effect on DNA demethylation by Tet1.

References

Roth M, Jeltsch A. Biotin-avidin microplate assay for the quantitative analysis of enzymatic methylation of DNA by DNA methyltransferases. Biol Chem 2000; 381:269-272.

Chih-Lin Hsieh, In vivo activity of murine de novo methyltransferases, Dnmt3a and Dnmt3b. Molecular and cellular biology 1999;19(12):8211-8.
